# ETV1 Drives CD4^+^ T Cell‐Mediated Intestinal Inflammation in Inflammatory Bowel Disease Through Amino Acid Transporter Slc7a5

**DOI:** 10.1002/advs.202511595

**Published:** 2025-12-05

**Authors:** Yan Shi, Song Wang, Yuqing Yan, Wei Chen, Yingying Cao, Dafan Chen, Wei Chen, Caiyun Ma, Xinjian Wan

**Affiliations:** ^1^ Digestive Endoscopic Center Shanghai Sixth People's Hospital Affiliated to Shanghai Jiao Tong University School of Medicine Shanghai 200233 China; ^2^ Department of Gastroenterology The Shanghai Tenth People's Hospital Tongji University School of Medicine Shanghai 200072 China; ^3^ Department of Gastroenterology Shanghai Sixth People's Hospital Affiliated to Shanghai Jiao Tong University School of Medicine Shanghai 200233 China

**Keywords:** ETV1, inflammatory bowel diseases, SLC7A5, Th17

## Abstract

Excessive CD4^+^ T cell responses drive inflammatory bowel disease (IBD), yet the transcriptional mechanisms underlying their dysfunction remain incompletely understood. Here, it is demonstrated that E‐twenty‐six variant transcription factor 1 (ETV1) is upregulated in IBD patients and positively correlates with disease severity. Etv1 deficiency impairs CD4^+^ T cell activation, proliferation, and T helper 17 (Th17) cell differentiation, thereby ameliorating TNBS‐induced colitis. Moreover, Etv1 deficiency attenuates CD45RB^high^CD4^+^ T cell‐induced colitis, characterized by a reduction in pathogenic CD4^+^ T cells in the intestinal mucosa. Pharmacological inhibition of ETV1 ameliorates colitis in recombination activating gene 1‐deficient mice and suppresses human IBD T cell responses ex vivo. Mechanistically, Etv1 binds to the promoter of the gene encoding the amino acid transporter solute carrier family 7 member 5 (Slc7a5), enhancing its expression and subsequent amino acid uptake to fuel T cell pathogenicity. Restoring Slc7a5 expression rescues the proliferation, differentiation, and colitogenic function of Etv1‐deficient CD4⁺ T cells. Clinically, SLC7A5 is upregulated in IBD, and its blockade ameliorates T cell‐driven colitis in vivo. Collectively, the results establish a critical role for the ETV1‐Slc7a5 axis in driving pathogenic CD4⁺ T cell responses in IBD, highlighting this pathway as a novel therapeutic target.

## Graphical abstract

This study identifies the transcription factor ETV1 as a key driver of CD4⁺ T cell‐mediated intestinal inflammation in inflammatory bowel disease (IBD). ETV1 promotes CD4⁺ T cell activation, proliferation, and Th17 differentiation by activating the amino acid transporter SLC7A5, fueling metabolic reprogramming. Inhibition of the ETV1‐SLC7A5 axis ameliorates CD4⁺ T cell‐mediated colitis, highlighting its therapeutic potential.

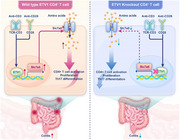

## 1 Introduction

Inflammatory bowel diseases (IBD), mainly including ulcerative colitis (UC) and Crohn's disease (CD), are chronic inflammatory disorders of the gastrointestinal tract.^[^ ^1^, ^2^ ^]^ A hallmark of IBD is the excessive infiltration of immune cells (e.g., T cells, B cells, and macrophages) into the intestinal mucosa, accompanied by the elevated production of pro‐inflammatory factors.^[^ ^3^, ^4^, ^5^ ^]^ Although the exact pathology and etiology of IBD remain elusive, it is well‐established that aberrantly activated CD4^+^ T cells are pivotal drivers of disease pathology.^[^ ^6^ ^]^ Their rapid proliferation and differentiation into effector subsets, such as T helper 1 (Th1) and Th17 cells, are central to disease progression.^[^ ^7^, ^8^, ^9^, ^10^ ^]^

The activation of naïve CD4⁺ T cells, initiated by T cell receptor (TCR) engagement, is the critical first step in adaptive immune responses, which can aberrantly target self‐tissues in autoimmune conditions.^[^ ^11^ ^]^ This transition from a quiescent to an activated state entails immense biosynthetic demands. To divide and execute effector functions, T cells must drastically increase their biomass, a process reliant on the abundant uptake of extracellular amino acids as fundamental building blocks for protein and nucleic acid synthesis. Accordingly, the expression of amino acid transporters on the T cell surface can surge by up to 100‐fold upon activation.^[^ ^12^ ^]^ Although the functions of many amino acid transporters remain incompletely understood, certain solute carrier (SLC) family members are well‐established in immunity. Among the SLC family, solute carrier family 7 member 5 (SLC7A5) is a key regulator of the multi‐step process of CD4⁺ T cell responses, from initial activation to effector function.^[^ ^13^, ^14^, ^15^, ^16^, ^17^ ^]^ However, the upstream transcriptional regulation of SLC7A5, especially within the context of T cell immunity and IBD, remains poorly defined.

E‐twenty‐six variant transcription factor 1 (ETV1), a member of the PEA3 subfamily, binds to DNA via a conserved winged helix‐turn‐helix domain recognizing a 5′‐GGA(A/T)‐3′ core sequence. To date, research on the function of ETV1 has primarily focused on the fields of cancer biology, fibrosis, and organ development.^[^ ^18^, ^19^, ^20^ ^]^ However, the roles of ETV1 in the immune system have long been ignored.

In this study, we identify ETV1 as a key driver of pathogenic CD4⁺ T cell responses in IBD. We demonstrate that ETV1, which is upregulated in CD4⁺ T cells from IBD patients, is essential for T cell activation, proliferation, and Th17 cell differentiation in vitro and in murine colitis models. Mechanistically, ETV1 regulates Slc7a5 to fuel the amino acid metabolism that sustains these effector functions. Consequently, pharmacological inhibition of either ETV1 or SLC7A5 ameliorated T cell‐driven colitis. Our work thus defines the ETV1‐SLC7A5 axis as a central immunometabolic pathway and a promising therapeutic target in IBD.

## 2 Results

### 2.1 ETV1 Is Upregulated in IBD and Positively Correlates with Disease Severity

**Figure 1:**
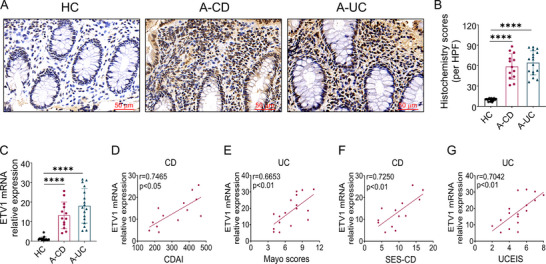
ETV1 expression is upregulated in IBD and positively correlates with disease activity. A–C) Two colonoscopic biopsy tissues were collected from each patient with active UC (A‐UC, n = 17), patients with active CD (A‐CD, n = 13), and healthy controls (HC, n = 17) and subjected to ETV1 immunohistochemistry and qRT‐PCR analysis, respectively. A) Representative immunohistochemical images of ETV1 staining. B) Quantification of ETV1 protein level by histochemistry score. C) qRT‐PCR analysis of ETV1 mRNA level. D) Correlation analysis of ETV1 mRNA level with the CDAI in patients with active CD. E) Correlation analysis of ETV1 mRNA expression with the Mayo scores in patients with UC. F) Correlation analysis of ETV1 mRNA expression with SES‐CD in patients with CD. G) Correlation analysis of ETV1 mRNA expression with UCEIS in patients with UC. Data are expressed as mean ± SEM. Statistical analyses were performed by one‐way ANOVA followed by Dunnett's multiple comparisons test (B,C) and Pearson's correlation (D–G). ^✱✱✱✱^ *p* < 0.0001. A‐CD, active Crohn's disease; A‐UC, active ulcerative colitis; CDAI, Crohn's Disease Activity Index; SES‐CD, Simple Endoscopic Score for Crohn's Disease; UCEIS, Ulcerative Colitis Endoscopic Index of Severity; HPF, high power field; ANOVA, analysis of variance.

To explore the relationship between ETV1 and IBD, inflamed colonic tissues were collected from patients with active IBD and the expression of ETV1 was detected by immunohistochemistry and quantitative reverse transcription polymerase chain reaction (qRT‐PCR). Immunohistochemical staining revealed that protein levels of ETV1 were markedly elevated in the inflamed mucosa from patients with IBD (**Figure** **1**A,B). Consistently, qRT‐PCR analysis showed that mRNA levels of ETV1 exhibited a significant increase in the inflamed colon tissues of patients with IBD compared with those in healthy controls (Figure 1C). Next, the correlation of colonic ETV1 mRNA level and clinical disease characteristics in IBD was analyzed. Surprisingly, a positive correlation was found between the Crohn's Disease Activity Index (CDAI) and ETV1 expression in active CD patients (Figure 1D), and between the Mayo scores and ETV1 expression in active UC patients (Figure 1E). Similarly, colonic ETV1 mRNA expression was positively correlated with the Simple Endoscopic Score for Crohn's Disease (SES‐CD) in active CD patients (Figure 1F), and with the Ulcerative Colitis Endoscopic Index of Severity (UCEIS) in active UC patients (Figure 1G). Collectively, our findings indicate that elevated ETV1 expression is associated with aggressive disease phenotypes in IBD, implicating its potential role in driving intestinal inflammation.

### 2.2 Etv1 Is Dispensable for Immune Homeostasis but Essential for Amplifying Intestinal Inflammation

**Figure 2:**
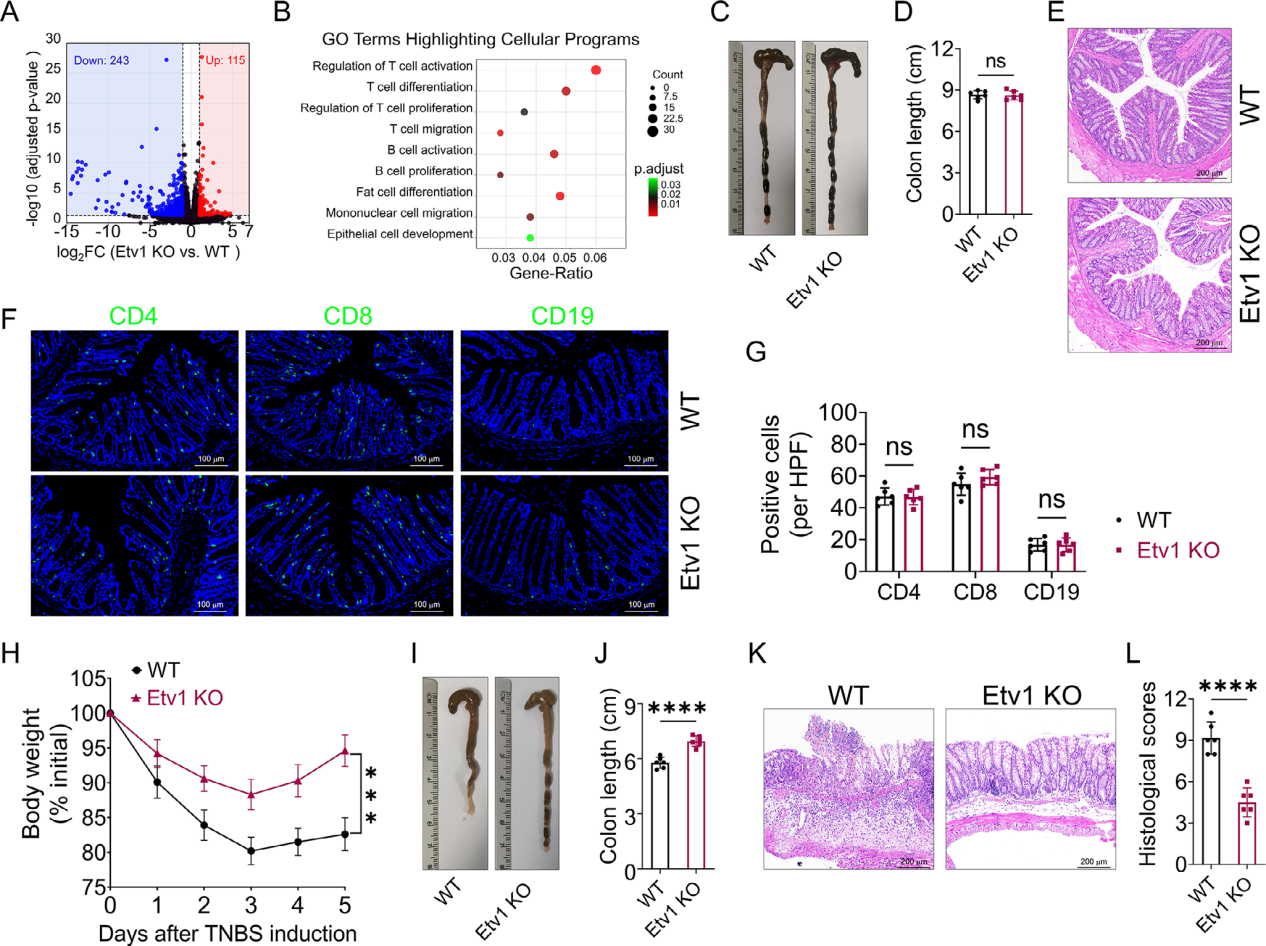
Etv1 is dispensable for homeostasis but critical for TNBS‐induced colitis. A,B) Colonic RNA from Etv1 KO and WT mice under steady‐state conditions was subjected to RNA‐sequencing analysis (n = 3 biological replicates per group). A) Volcano plot highlighting differentially down‐regulated genes (blue) and up‐regulated genes (red) with a cutoff at |log_2_FC| ≥ 0.5 and adjusted p‐value < 0.05. B) Gene Ontology (GO) enrichment analysis for cellular programs. C–G) Colons from 8‐week‐old Etv1 KO and WT mice under steady‐state conditions were evaluated. C) Representative gross morphology and D) quantified length of colons. E) H&E staining of colon sections. F) Immunofluorescence staining for CD4 (left panels), CD8 (middle panels), and CD19 (right panels) in colonic sections. G) Quantification of CD4^+^ T cells, CD8^+^ T cells, and CD19^+^ B cells. H‐L) TNBS‐induced acute colitis was performed in Etv1 KO (n = 6) and WT (n = 6) mice, and colonic tissues were collected on day 5 after TNBS exposure. H) Mouse body weight changes. I) Representative gross morphology and J) colon length. K) Representative colonic H&E staining and L) histological scores. ^✱✱✱^ *p* < 0.001, ^✱✱✱✱^ *p* < 0.0001. (C–L) Data are pooled from three independent experiments and are expressed as mean ± SEM. Statistical analysis was evaluated using Unpaired Student's t‐test (D,G,H,J) or Mann–Whitney U test (L). KO, knockout; WT, wild type; H&E, hematoxylin and eosin; TNBS, 2,4,6‐trinitrobenzenesulfonic acid; CD4, cluster of differentiation 4; CD8, cluster of differentiation 8; CD19, cluster of differentiation 19; ns, no significance.

To investigate the function of ETV1 in IBD, we generated Etv1 knockout (KO) mice and validated the gene deletion (Figure S1A, Supporting Information). We first assessed the global transcriptional impact of Etv1 deficiency by performing bulk RNA‐sequencing (seq) of steady‐state colon tissues from Etv1 KO and wild‐type (WT) littermates, and identified 243 down‐regulated and 115 up‐regulated genes (**Figure** **2**A). Gene Ontology (GO) enrichment analysis revealed a broad spectrum of significantly enriched terms, most of which were involved in immune system processes (Figure S2, Supporting Information). A focused analysis showed that this immune signature comprised a dysregulation of genes governing key cellular programs, including the activation, proliferation, and differentiation of T and B cells, alongside mononuclear cell migration and epithelial cell development (Figure 2B; Table S1, Supporting Information). This transcriptomic profile suggested a potential role for Etv1 in modulating immune and structural programs in the colon. We next asked whether these transcriptional changes would translate to alterations in cellular homeostasis. Under steady‐state conditions, the macroscopic and microscopic architecture of the colon in Etv1 KO mice was normal (Figure 2C–E). Consistent with this, immunofluorescence staining revealed comparable numbers of CD4⁺ and CD8⁺ T cells and CD19⁺ B cells in the colon (Figure 2F,G). The frequencies of Ifng⁺, Il4⁺, Il17a⁺, Foxp3⁺, and Il10⁺ cells among lamina propria CD4⁺ T cells were unaltered (Figure S1B,C, Supporting Information). Furthermore, the cellularity of spleen and mesenteric lymph nodes, as well as the proportions of major immune cell subsets (e.g., T cells, dendritic cells, NK cells, and B cells) within spleens, were also unaffected by Etv1 deficiency (Figure S3, Supporting Information). Collectively, although Etv1 deletion perturbs the transcriptional landscape of immune‐related pathways, it is dispensable for immune homeostasis under steady‐state conditions.

We therefore next investigated whether Etv1 deficiency affects the intestinal immune response under inflammatory conditions. To this end, we established a 2,4,6‐trinitrobenzene sulfonic acid (TNBS)‐induced acute colitis model in Etv1 KO and WT mice, and monitored body weight daily. Etv1 KO mice exhibited significantly less body weight loss (Figure 2H) and colon shortening (Figure 2I,J) compared to WT mice. Importantly, ETV1 deficiency significantly improved TNBS‐induced colitis, as characterized by reduced lymphocyte infiltration and lower histopathological scores in colon tissue (Figure 2K,L). Thus, ETV1 is not required for maintaining immune homeostasis under steady‐state conditions but is essential for amplifying intestinal inflammation.

### 2.3 Etv1 Deficiency Alleviates CD4^+^ T Cell Responses in TNBS‐Induced Colitis

Our initial immunohistochemical analysis of colonic tissues from IBD patients indicated a broad cellular distribution of ETV1 expression within inflamed areas, involving both epithelial and stromal compartments (Figure 1A). As previously described,^[^ ^6^, ^21^ ^]^ uncontrolled activation and expansion of pro‐inflammatory CD4^+^ T cells in the intestinal lamina propria play a central role in the pathogenesis of IBD. We therefore sought to determine whether ETV1 is enriched in colonic CD4⁺ T cells and participates in CD4⁺ T cell‐mediated immune response in IBD. We first examined ETV1 expression in CD4⁺ T cells infiltrating the inflamed colonic mucosa of patients with active IBD using immunofluorescence staining. As shown in Figure S4 (Supporting Information), we observed a significant accumulation of ETV1^+^CD4^+^ T cells in inflamed tissues from patients with active CD and UC compared to healthy controls, supporting a strong association between ETV1 and CD4^+^ T cell immune responses in IBD. Given that CD4⁺ T cells in the inflamed mucosa are persistently activated, we asked whether TCR signaling drives ETV1 upregulation. To test this, we stimulated purified CD4⁺ T cells from healthy donors with anti‐CD3/CD28 antibodies at different doses (2 or 5 µg mL^−1^) for varying durations. We found that ETV1 expression was induced in a time‐dependent manner (Figure S5A,B, Supporting Information). However, the induction was significantly attenuated at the higher concentration compared to the lower dose (Figure S5C–E, Supporting Information). These findings suggest that ETV1 might contribute to CD4⁺ T cell‐driven immune responses in IBD.

**Figure 3:**
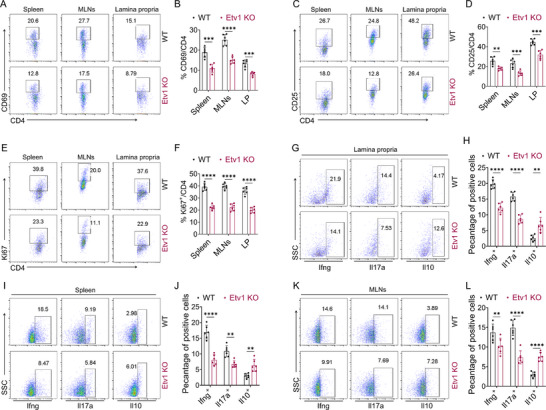
Etv1 deficiency alleviates CD4^+^ T cell responses in TNBS‐induced colitis. A–F) TNBS‐induced colitis was performed in Etv1 KO (n = 6) and WT (n = 6) mice, and mice were euthanized on day 2 after TNBS exposure. CD4^+^ T cells in spleens, mesenteric lymph nodes (MLNs), and intestinal lamina propria (LP) were isolated for A,B) CD69, C,D) CD25 and E,F) Ki67 staining by flow cytometry. Quantification of CD4^+^CD69^+^ T cells, CD4^+^CD25^+^ T cells, and CD4^+^Ki67^+^ cells are shown in the bar charts. G–K) TNBS‐induced colitis was performed in Etv1 KO (n = 6) and WT (n = 6) mice, and mice were euthanized on day 5 after TNBS exposure. CD4^+^ T cells were isolated from the spleens, MLNs, and intestinal lamina propria, and the intracellular expression of Ifng, Il17a, and Il10 was detected by flow cytometry. Bar charts show the percentages of Ifng^+^, Il17a^+^, and Il10^+^ cells. ^✱✱^ *p* < 0.01, ^✱✱✱^ *p* < 0.001, ^✱✱✱✱^ *p* < 0.0001. All data are pooled from three independent experiments and are expressed as mean ± SEM. Statistical analysis was evaluated using an unpaired Student's t‐test. KO, knockout; WT, wild type; TNBS, 2,4,6‐trinitrobenzenesulfonic acid; MLNs, mesenteric lymph nodes; LP, intestinal lamina propria; CD4, cluster of differentiation 4; CD69, cluster of differentiation 69; CD25, cluster of differentiation 25; IL, interleukin; SSC, side scatter; IFN, interferon.

To directly test this hypothesis, we evaluated the cell‐associated responses of CD4^+^ T cells from Etv1 KO mice during colitis. Given the impact of Etv1 deficiency on genes involved in T cell activation, proliferation, and differentiation (Figure 2B), we first assessed the impact of Etv1 deficiency on T cell activation and proliferation during the early phase (day 2) of TNBS‐induced colitis. CD4^+^ T cells were isolated from the intestinal lamina propria, spleens, and MLNs on day 2 after TNBS challenge. Flow cytometric analysis revealed that CD4^+^ T cells from Etv1 KO mice exhibited reduced expression of the early activation marker CD69 (**Figure** **3**A,B) and the late activation marker CD25 (Figure 3C,D), as well as a significant decrease in the proportion of Ki67⁺ proliferating cells (Figure 3E,F). To determine whether the early impairment in T cell activation would translate to sustained changes in effector function, we further profiled the intracellular expression of effector cytokines in CD4⁺ T cells on day 5 post‐TNBS exposure. We found that Etv1 KO mice exhibited significantly lower frequencies of Ifng⁺ and Il17a⁺ cells, coupled with an increase in anti‐inflammatory Il10⁺ cells, among CD4⁺ T cells from colon, spleen, and MLNs (Figure 3G–L). Collectively, these data demonstrate that Etv1 deficiency attenuates CD4⁺ T cell activation, proliferation, and pro‐inflammatory effector responses while promoting an anti‐inflammatory capacity, thereby ameliorating CD4⁺ T cell‐mediated colitis.

### 2.4 Etv1 Deficiency Impairs CD4^+^ T Cell Activation, Proliferation and Th17 Differentiation in vitro

**Figure 4:**
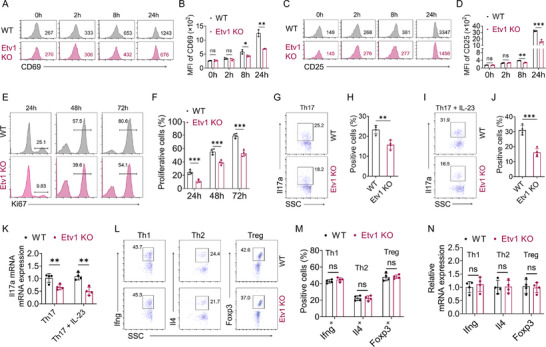
Etv1 deficiency disturbs CD4^+^ T cell activation, proliferation, and Th17 cell differentiation in vitro. A–F) Splenic CD4^+^ T cells (5×10^5^/well) of the indicated genotypes were activated with anti‐CD3/28 antibodies in vitro for the indicated times (n ≥ 3 per genotype). A, C) Representative flow cytometric analysis and B,D) mean fluorescence intensity (MFI) of activation markers CD69 and CD25 (n = 3 per genotype). E) Representative flow cytometric analysis and F) percentage of the indicated proliferating CD4^+^ T cells labeled with Ki67 (n = 4 per genotype). G–J) Flow cytometric analysis of Il17a expression in CD4^+^ T cells of indicated genotypes cultured under Th17 conditions (TGF‐β + IL‐6) with (I,J) or without (G,H) IL‐23 for 5 days (n = 4 per genotype). K) qRT‐PCR analysis of Il17a mRNA expression in the indicated differentiation assays (n = 4 per genotype). L,M) Analysis of Ifng⁺, Il4⁺, and Foxp3⁺ cell populations in differentiated CD4⁺ T cells by flow cytometry under the indicated culture conditions (n = 4 per genotype). N) qRT‐PCR analysis of Ifng, Il4 Foxp3 in the indicated differentiation condition (n = 4 per genotype). ^✱^ *p* < 0.05, ^✱✱^ *p* < 0.01, ^✱✱✱^ *p* < 0.001. All data are pooled from three independent experiments and are expressed as mean ± SEM. Statistical analysis was evaluated using an unpaired Student's t test. MFI, mean fluorescence intensity; ns, no significance; CD69, cluster of differentiation 69; CD25, cluster of differentiation 25; TGF, transforming growth factor; Il, interleukin; Th, T helper; Ifn, interferon; SSC, side scatter; Foxp3, Forkhead box protein P3; Treg, Regulatory T cells.

Building on these findings, we next investigated whether Etv1 intrinsically regulates CD4⁺ T cell immune responses in vitro. To evaluate the role of Etv1 in T cell activation and proliferation, splenic CD4⁺ T cells were isolated and stimulated with anti‐CD3/CD28 antibodies for the indicated time points. Flow cytometric analysis showed that Etv1‐deficient CD4⁺ T cells exhibited reduced expression of CD69 (**Figure** **4**A,B) and CD25 (Figure 4C,D). Further, Ki67 staining revealed a significant reduction in the proportion of proliferating Etv1‐deficient CD4⁺ T cells following activation (Figure 4E,F).

Having established the cell‐intrinsic role of Etv1 in T cell activation and proliferation, we then asked whether it also governs CD4^+^ T cell differentiation. Naïve CD4^+^ T cells from Etv1 KO and WT mice were polarized in vitro to generate major effector and regulatory CD4⁺ T cell subsets. Under both non‐pathogenic Th17 (TGF‐β + IL‐6) and pathogenic Th17 (TGF‐β + IL‐6 + IL‐23) polarizing conditions, Etv1 KO cells generated much lower frequencies of Il17a⁺ cells compared to WT controls (Figure 4G–J). This impairment was corroborated at the transcriptional level, with significantly reduced Il17a mRNA expression under both Th17‐polarizing conditions (Figure 4K). In contrast to its effect on Th17 differentiation, Etv1 deficiency did not alter the frequencies of Ifng⁺ (Th1), Il4⁺ (Th2), and Foxp3⁺ (Treg) cells under their respective polarizing conditions (Figure 4L,M). Consistently, no significant differences were observed in the mRNA expression of Ifng (Th1), Il4 (Th2), and Foxp3 (Treg) in Etv1 KO cells under their respective conditions (Figure 4N). Collectively, these in vitro findings establish an essential, cell‐intrinsic role for Etv1 in CD4⁺ T cell activation, proliferation, and specifically, in Th17 lineage commitment.

### 2.5 Etv1 Deficiency Intrinsically Attenuates CD4^+^ T Cell‐Mediated Colitis In Vivo

**Figure 5:**
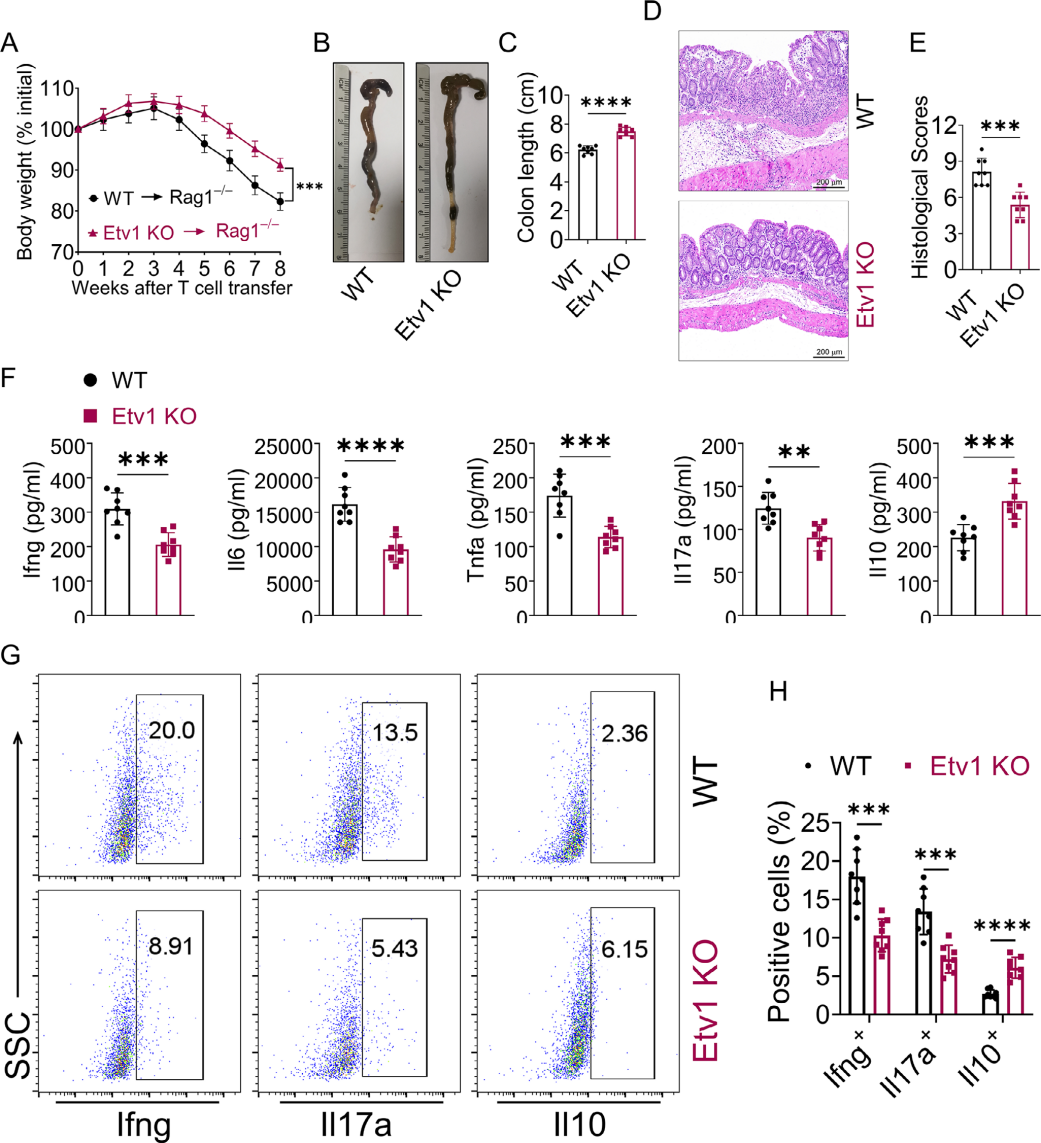
Etv1‐deficient CD45RB^high^CD4^+^ T cells are defective in the induction chronic colitis in Rag1^−/−^ mice. WT and Etv1‐deficient CD45RB^high^CD4^+^ T cells were intraperitoneally transferred to Rag1^−/−^ mice (n = 8 per group, 5×10^5^ cells per mouse). A) Colitis‐induced weight change. B) Gross morphology and C) length of the large bowels from Rag1^−/−^ mice 8 weeks after adoptive transfer. D) Representative images of H&E‐stained colon sections and E) histopathological analysis. F) Cytokine levels in the colon. G) Representative flow cytometric images and H) the frequencies of Ifng^+^, Il17a^+^, and Il10^+^ T cells isolated from the intestinal lamina propria. ^✱✱^ *p* < 0.01, ^✱✱✱^ *p* < 0.001, ^✱✱✱✱^ *p* < 0.0001. All data are pooled from three independent experiments and are expressed as mean ± SEM. Statistical analysis was evaluated using unpaired Student's t‐test (A, C, F, H) or Mann–Whitney U test (E). Il, interleukin; Ifn, interferon; Tnf, tumor necrosis factor; Rag, recombination activation gene; H&E, hematoxylin and eosin; SSC, side scatter; KO, knockout; WT, wild type; TNBS, 2,4,6‐trinitrobenzenesulfonic acid; CD4, cluster of differentiation 4.

To directly probe the cell‐intrinsic role of Etv1 in CD4^+^ T cell‐driven intestinal inflammation, CD45RB^high^ CD4^+^ T cells sorted from Etv1 KO or WT mice were intraperitoneally transferred into recombination activating gene 1‐deficient (Rag1^−/−^) mice. Recipients of WT T cells exhibited markedly greater body weight loss than those receiving Etv1 KO CD4⁺ T cells (**Figure** **5**A). At the macroscopic level, colons from mice reconstituted with Etv1 KO T cells exhibited significantly less shortening, edema, and wall thickening than those from controls (Figure 5B,C). Histological assessment revealed substantially reduced lymphocytic infiltration, epithelial hyperplasia, goblet cell depletion, and ulceration in mice receiving Etv1‐deficient T cells compared to those receiving WT T cells (Figure 5D), resulting in significantly lower histological scores (Figure 5E). Furthermore, Etv1 deficiency resulted in significantly reduced levels of pro‐inflammatory cytokines (e.g., Ifng, Il6, Tnfa, Il17a) and an elevated level of the anti‐inflammatory cytokine Il10 in the colon (Figure 5F). Consistent with the ameliorated tissue immune pathology, mice that received Etv1‐deficient T cells showed significantly lower Il17a^+^ and Ifng^+^ cells in colon‐infiltrating CD4^+^ T cells (Figure 5G,H). In contrast, the proportion of Il10^+^ cells was increased in colon‐infiltrating CD4^+^ T cells (Figure 5G,H). Thus, our findings demonstrate that Etv1 intrinsically amplifies pro‐inflammatory effector functions while restraining anti‐inflammatory capacity in CD4⁺ T cells, thereby driving colitis.

### 2.6 Pharmacological Inhibition of Etv1 Ameliorates T Cell‐Mediated Colitis

**Figure 6:**
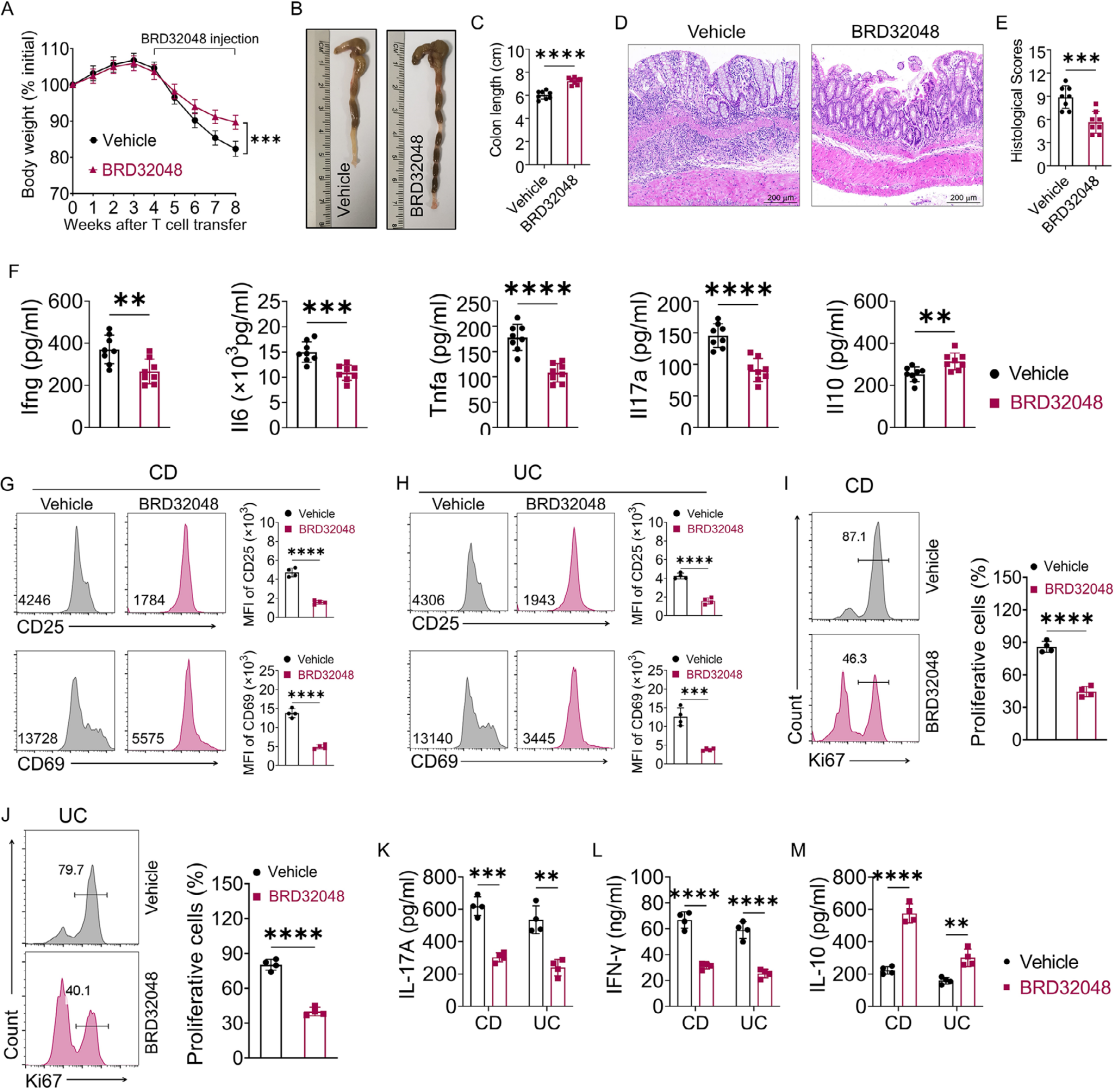
ETV1 inhibitor alleviates colitis in Rag1^−/−^ recipients, and regulates the effector function of T cells from IBD patients. A–F) WT CD45RB^high^CD4^+^ T cells were sorted and adoptively transferred intraperitoneally into Rag1^−/− ^recipient mice (5×10^5^ cells per mouse). Mice were treated with or without ETV1 inhibitor BRD32048 (n = 8 per group) every other day from 4 weeks post cell transfer for an additional 4 weeks. A) The weight change in each group during the experiment. B) Gross morphology and C) length of colons. D,E) Colitis severity was assessed by D) H&E staining and E) histological scores. F) Colonic secretion of cytokines in each group. G–M) Peripheral blood CD4^+^ T cells from patients with active CD (n = 4) and UC (n = 4) were stimulated with anti‐CD3/CD28 antibodies in the presence of BRD32048. G, H) Expression of activation markers CD25 (upper panels) and CD69 (lower panels) was detected by flow cytometry at 24 h. I,J) Cell proliferation was assessed by flow cytometric analysis of Ki67 staining at 72 h. K–M) Cytokine levels (IL‐17A, IFN‐γ, IL‐10) in culture supernatants were measured by ELISA on day 5. ^✱✱^ *p* < 0.01; ^✱✱✱^ *p* < 0.001; ^✱✱✱✱^ *p* < 0.0001. All data are pooled from three independent experiments and are expressed as mean ± SEM. Statistical analysis was evaluated using unpaired Student's t‐test (A,C,F,G–M) or Mann–Whitney U test (E). IL, interleukin; IFN, interferon; Rag1, recombination activation gene1; H&E, hematoxylin and eosin; CD, Crohn's disease; UC, ulcerative colitis.

Having shown that genetic ETV1 deletion intrinsically ameliorates CD4⁺ T cell‐driven colitis, we next asked whether pharmacological inhibition of ETV1 could achieve a similar therapeutic effect. To this end, we evaluated BRD32048, a selective small‐molecule inhibitor previously shown to promote Etv1 degradation and regulate cellular function,^[^ ^22^ ^]^ in the WT CD4⁺ T cell transfer‐induced colitis model. Recipient mice received intraperitoneal injections of either BRD32048 or vehicle control every other day for four weeks, beginning 4 weeks after T cell transfer. BRD32048 treatment significantly ameliorated the severity of colitis, as evidenced by attenuated body weight loss (**Figure** **6**A), reduced colon shortening (Figure 6B,C), and improved histopathology with diminished lymphocytic infiltration (Figure 6D) and lower histopathological scores (Figure 6E). Concurrently, we observed a shift in the cytokine profile, characterized by decreased pro‐inflammatory cytokines (Ifng, Il6, Tnfa, and Il17) and increased anti‐inflammatory Il0 in the colon (Figure 6F). These results demonstrate that pharmacological inhibition of Etv1 effectively treats ongoing T cell‐mediated colitis.

To further establish the therapeutic potential and specificity of BRD32048, we assessed its potential toxicity in healthy, non‐colitic mice. H&E staining analysis confirmed normal colonic architecture without significant immune infiltration (Figure S6A, Supporting Information). Moreover, serum biochemical analysis showed that levels of alanine aminotransferase (ALT), aspartate aminotransferase (AST), blood urea nitrogen (BUN), and creatinine (CRE) were all within normal ranges and comparable across groups (Figure S6B, Supporting Information). Collectively, these data indicate that BRD32048 exhibits no detectable toxicity in healthy mice.

### 2.7 Targeting ETV1 Suppresses Pathogenic Human CD4⁺ T Cell Responses in IBD

We next examined the effect of ETV1 inhibition in human CD4⁺ T cells from IBD patients. To this end, CD4^+^ T cells were isolated from peripheral blood mononuclear cells (PBMCs) of IBD patients and activated with anti‐CD3/CD28 antibodies in the presence or absence of the ETV1 inhibitor BRD32048. Flow cytometric analysis showed that BRD32048 treatment significantly reduced CD69 and CD25 expression (Figure 6G,H) in CD4^+^ T cells cultured for 24 h. Ki67 staining demonstrated that the proportion of proliferating cells was markedly reduced following BRD32048 treatment (Figure 6I,J). Importantly, BRD32048 treatment markedly suppressed the production of IL‐17A (Figure 6K) and IFN‐γ (Figure 6L) while enhancing IL‐10 production (Figure 6M). Collectively, these results demonstrate that pharmacological inhibition of ETV1 with BRD32048 effectively suppresses human pathogenic T cell responses, highlighting its potential therapeutic value for IBD.

### 2.8. Amino Acid Transporter Slc7a5 Is a Target of Etv1 and a Key Factor in Regulating Intestinal Inflammation

**Figure 7:**
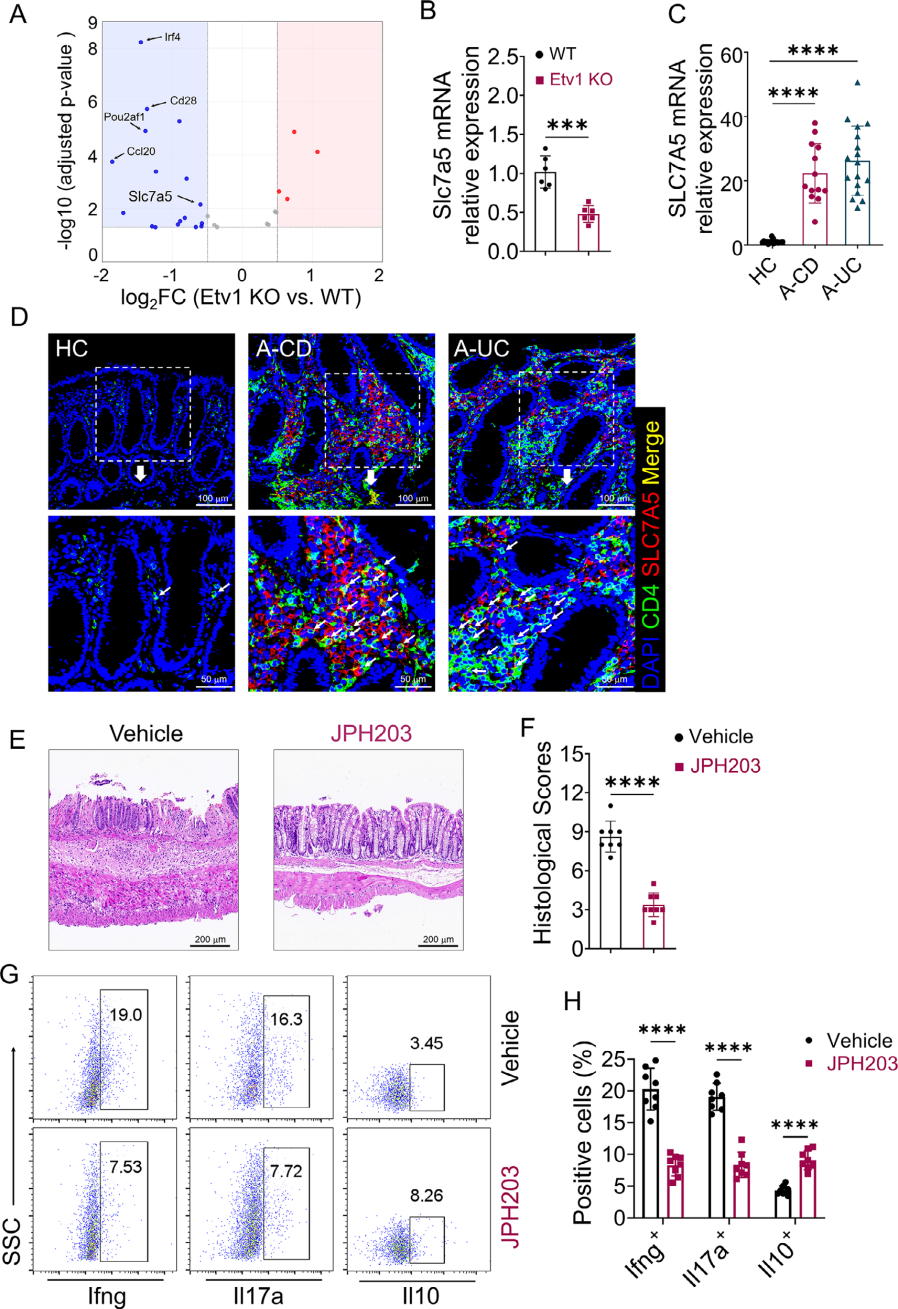
Slc7a5 is a target of Etv1 and a key factor in regulating intestinal inflammation. A) Volcano plot following GO analysis of the differentially expressed genes identified in Figure 2A, highlighting those associated with the positive regulation of cytokine production pathway (red, upregulated, blue, downregulated; |log_2_FC| ≥ 0.5, adjusted p‐value < 0.05). B) Splenic CD4^+^ T cells from WT and Etv1‐deficient mice were activated with anti‐CD3/CD28 antibodies for 48 h, and the mRNA level of Slc7a5 was detected by qRT‐PCR. C, D) Colonic biopsies from the same patient cohort described in Figure 1A were analyzed to determine SLC7A5 mRNA expression and SLC7A5^+^CD4^+^ T cell infiltration (HC, n = 17; A‐CD, n = 13; A‐UC, n = 17). C) SLC7A5 mRNA expression measured by qRT‐PCR. D) Representative immunofluorescence images stained for DAPI (blue), CD4 (green), and SLC7A5 (red). White arrows indicate SLC7A5^+^CD4^+^ T cells. E‐H) WT CD45RB^high^CD4^+^ T cells were sorted and adoptively transferred into Rag1^−/−^ mice (5×10^5^ cells per mouse, n = 8 per group). Starting on the day of T cell transfer, recipient mice were treated with JPH203 or vehicle control every other day until sacrifice at 8 weeks. E) Representative H&E‐stained sections of colon tissues. F) Histological scores of colitis. G) Representative flow cytometry plots and H) quantitative summary of the frequencies of Il17a⁺, Ifng⁺, and Il10⁺ cells among intestinal lamina propria CD4⁺ T cells. ^✱✱✱^ *p* < 0.001,^✱✱✱✱^ *p* < 0.0001. (B‐H) Data are pooled from three independent experiments and are expressed as mean ± SEM. Statistical analysis was evaluated using unpaired Student's t‐test (B, H) or one‐way ANOVA followed by Dunnett's multiple comparisons test (C) or Mann–Whitney U test (F). SLC7A5, solute carrier family 7 member 5; Il, interleukin; Ifn, interferon; Rag1, recombination activation gene1; H&E, hematoxylin and eosin; HC, healthy control; A‐CD, active Crohn's disease; A‐UC, active ulcerative colitis; CD4, cluster of differentiation 4; DAPI, 4′,6‐diamidino‐2‐phenylindole.

To define the mechanism by which Etv1 regulates T cell effector functions, we re‐analyzed our RNA‐seq data (Figure 2A) for candidate targets. GO analysis identified the positive regulation of the cytokine production pathway as significantly enriched among dysregulated genes (**Figure** **7**A). Notably, Slc7a5, an amino acid transporter with established roles in T cell activation, proliferation, and Th17 differentiation,^[^ ^14^, ^16^ ^]^ is among the most markedly down‐regulated transcripts in this pathway (Figure 7A). Given this conserved role and its position as a downstream target of ETV1, we hypothesized that Slc7a5 mediates Etv1‐driven pathology in IBD. We first confirmed that Slc7a5 expression was significantly reduced in Etv1‐deficient CD4⁺ T cells (Figure 7B). Assessment of its clinical relevance revealed that SLC7A5 mRNA expression was elevated in inflamed intestinal tissues from IBD patients (Figure 7C). Furthermore, immunofluorescence analysis of colonic sections indicated an increase in SLC7A5⁺CD4⁺ T cells within the actively inflamed mucosa (Figure 7D), suggesting a role for SLC7A5 in the pathogenesis of IBD through the regulation of T cell immune responses.

To functionally establish Slc7a5 as a key regulator in vivo, we treated WT CD45RB^high^CD4^+^ T cell‐transferred Rag1^−/−^ mice with the specific Slc7a5 inhibitor JPH203. JPH203 treatment markedly mitigated the severity of colitis, as evidenced by diminished lymphocytic infiltration (Figure 7E) and lower histological scores (Figure 7F). Flow cytometric analysis revealed that JPH203 treatment reduced the frequency of Il17a⁺ and Ifng⁺ cells while increasing that of Il10⁺ cells among colonic CD4⁺ T cells (Figure 7G,H). Collectively, these data identify Slc7a5 as a downstream target of the Etv1 and implicate it as a crucial driver of pathogenic CD4⁺ T cell responses in IBD.

### 2.8 Etv1 Regulates CD4^+^ T Cells Proliferation and Th17 Differentiation through Slc7a5 in IBD

**Figure 8:**
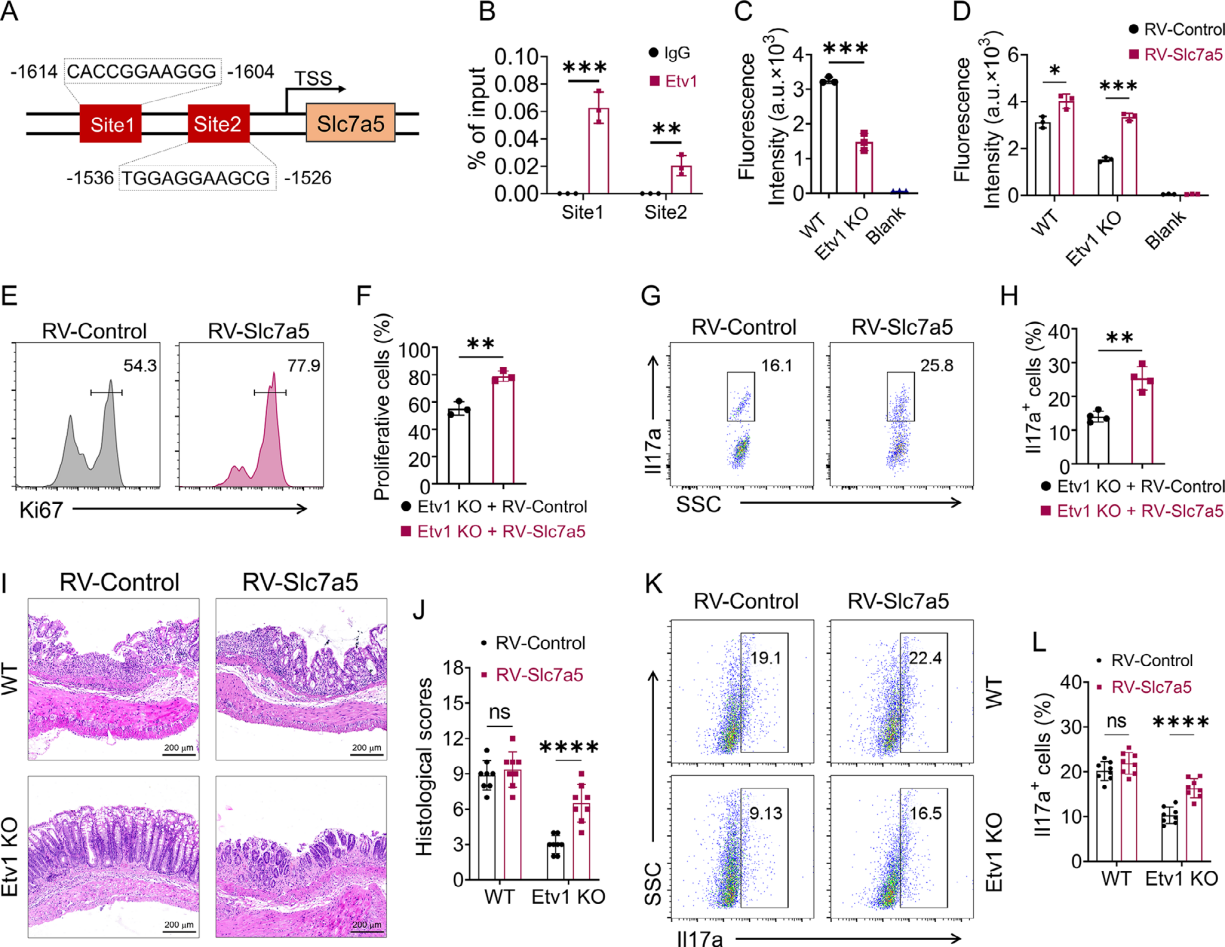
The regulatory role of Etv1 in CD4^+^ T cell response depends on Slc7a5. A) Schematic diagram of the putative Etv1 binding sites in the Slc7a5 promoter, identified via the JASPAR database. B) ChIP‐qPCR analysis of Etv1 enrichment at the putative binding sites in the Slc7a5 promoter, using an Etv1‐specific antibody. C) Amino acid uptake in WT and Etv1‐deficient CD4^+^ T cells activated with anti‐CD3/28 antibodies for 24 h in vitro (n = 3 per group). D) Amino acid uptake in WT and Etv1‐deficient CD4^+^ T cells transduced with control retrovirus (RV‐Control) or Slc7a5‐expressing retrovirus (RV‐Slc7a5) and cultured for an additional 24 h in vitro (n = 3 per group). E) Representative flow cytometric analysis and F) quantification of the proliferation of Etv1‐deficient CD4^+^ T cells transduced with RV‐Control or RV‐Slc7a5 cultured for a further 48 h (n = 3 per genotype) in vitro. G,H) Etv1‐deficient CD4⁺ T cells were transduced with RV‐Control or RV‐Slc7a5 and cultured under Th17‐polarizing conditions (TGF‐β + IL‐6 + IL‐23) for 4 days. G) Representative flow cytometry images and H) the frequency of Il17a⁺ cells are shown (n = 4 per genotype). I–L) WT or Etv1‐deficient CD45RB^high^CD4^+^ T cells transduced with RV‐Control or RV‐Slc7a5 were transferred to Rag1^−/−^ mice (n = 8 per group). Colon inflammation was evaluated by I) H&E staining and J) histological scores. K) Representative flow cytometry plots and L) the percentage of Il17a^+^ cells among CD4^+^ T cells isolated from the lamina propria. ^✱^ *p* < 0.05, ^✱✱^ *p* < 0.01, ^✱✱✱^ *p* < 0.001, ^✱✱✱✱^ *p* < 0.0001. (B‐L) Data are pooled from three independent experiments and are expressed as mean ± SEM. Statistical analysis was evaluated using unpaired Student's t‐test (B, C, D, F, H, and L) or Mann–Whitney U test (J). Il, interleukin; Rag1, recombination activation gene1; H&E, hematoxylin and eosin; SSC, side scatter; KO, knockout; WT, wild type; Slc7a5, solute carrier family 7 member 5; ns, no significance; RV, retrovirus.

We next defined the molecular mechanism by which Etv1 regulates Slc7a5. Bioinformatic screening of the JASPAR database predicted two potential Etv1 binding sites in the mouse Slc7a5 promoter (**Figure** **8**A). We next performed chromatin immunoprecipitation (ChIP) assays in CD4⁺ T cells and found that Etv1 binds to the predicted Slc7a5 promoter region, as evidenced by significant enrichment (Figure 8B). Given the role of Slc7a5 in amino acid transport to support protein synthesis during CD4⁺ T cell responses, we asked whether Etv1 deficiency impairs amino acid uptake, thereby compromising T cell effector functions. Consistent with the decreased expression of Slc7a5, the capacity of amino acid uptake was markedly decreased in Etv1 KO CD4^+^ T cells compared to WT controls (Figure 8C), whereas forced expression of Slc7a5 potently reversed this effect (Figure 8D). Importantly, Slc7a5 overexpression significantly increased the proportion of proliferating CD4^+^ T cells (Figure 8E,F) and Il17a^+^ cells in Etv1 deficient CD4^+^ T cells (Figure 8G,H), although Slc7a5 overexpression exerted no obvious effect on WT CD4^+^ T cells (Figure S7, Supporting Information). These data indicate that Slc7a5 is a functional target of Etv1 in CD4^+^ T cells.

Lastly, the CD4^+^ T cells transfer Rag1^−/−^ colitis model was used to verify the function of Etv1 in the regulation of Slc7a5 in vivo. CD4^+^ T cells from Etv1 deficient and WT mice were transduced with Slc7a5‐expressing retrovirus (RV‐Slc7a5) or control retrovirus prior to transfer. RV‐Slc7a5 transduction partially restored the ability of Etv1 deficient CD4^+^ T cells in the induction of colitis in terms of histopathology (Figure 8I,J), although RV‐Slc7a5 overexpression did not significantly enhance the severity of colitis induced by WT CD4^+^ T cells (Figure 8I,J). Consistently, the frequencies of Il17a^+^CD4^+^ T cells in the colonic tissues were markedly increased by overexpression of Slc7a5 in Etv1 deficient CD4^+^ T cells (Figure 8K,L). Altogether, these data demonstrate that Etv1 drives CD4⁺ T cell pathogenicity in IBD via regulating Slc7a5.

## 3 Discussion

The critical role of CD4⁺ T cells in inflammatory bowel disease (IBD) is well‐established,^[^ ^10^, ^23^ ^]^ yet the key transcriptional regulators governing their dysfunction remain incompletely defined. Here, we identify the transcription factor ETV1 as a key regulator of this process. We demonstrate that ETV1 is highly expressed in the inflamed colon of IBD patients, with its expression levels correlating with clinical severity, and is notably enriched within infiltrating CD4⁺ T cells. Furthermore, in vitro stimulation of human CD4⁺ T cells shows that ETV1 expression is induced by T cell receptor (TCR) activation in a time‐dependent manner. To functionally contextualize these findings, we proceeded to define the specific contributions of ETV1 to CD4⁺ T cell biology.

T cell activation, the initial step in adaptive immune responses, is pivotal for eliminating pathogen infections and inducing autoimmunity. TCR engagement triggers T cell proliferation and differentiation into effector cells capable of producing cytokines. A hallmark of IBD is the uncontrolled activation and expansion of CD4^+^ T cells producing pro‐inflammatory cytokines in the intestinal lamina propria.^[^ ^24^ ^]^ Similar phenomena are observed in the TNBS‐induced mouse colitis model.^[^ ^25^ ^]^ Our data demonstrate that naïve T cells express low level of ETV1, while T cell activation greatly induces ETV1 expression. Further, Etv1 deletion reduces activator marker CD69 and CD25 expression and dampens expansion of CD4^+^ T cells upon TCR stimulation in vitro. These defects were also evident in TNBS‐induced colitis in Etv1‐deficient mice in vivo. However, no significant differences were observed under steady‐state conditions. Importantly, ETV1 inhibition also blocks activation and expansion of IBD CD4^+^ T cells. Collectively, these results demonstrated that ETV1 is essential for CD4^+^ T cell activation and proliferation under inflammatory conditions.

Th17 cells have been widely thought to contribute to intestinal inflammation in the development of IBD through producing pro‐inflammatory cytokine IL‐17A.^[^ ^26^, ^27^ ^]^ Abundant evidence suggests that different cytokine milieus induce naïve CD4^+^ T cells to differentiate into non‐pathogenic or pathogenic Th17 cells in vitro and in vivo.^[^ ^28^, ^29^ ^]^ TGF‐β1 and IL‐6 jointly induce the differentiation of Th17 cells, which typically exhibit a non‐pathogenic phenotype characterized by high IL‐10 production and limited inflammatory activity.^[^ ^30^ ^]^ In contrast, exposure to IL‐23 and/or IL‐1β can reprogram these cells into a pathogenic state, marked by elevated expression of pro‐inflammatory mediators (e.g., IL‐17A, IFN‐γ, and GM‐CSF) alongside reduced anti‐inflammatory factors IL‐10.^[^ ^31^ ^]^ This transition highlights the critical role of the cytokine microenvironment in determining Th17 cell pathogenicity. Notably, in vivo‐derived Th17 cells display functional plasticity, exerting either non‐pathogenic or pathogenic effects depending on contextual signals.^[^ ^7^ ^]^ Intriguingly, IL‐10‐secreting Th17 subsets can mediate bystander immunosuppression, counteracting the activity of fully differentiated pathogenic Th17 populations.^[^ ^29^, ^32^ ^]^ In the present study, we found that Etv1 deletion suppresses the differentiation of both non‐pathogenic and pathogenic Th17 cells in vitro. Despite the functional dichotomy of Th17 cells, Etv1 deficiency mitigated both TNBS‐induced acute colitis and CD4⁺ T cell‐transfer chronic colitis in vivo. The attenuated disease was characterized not only by a reduction in colitogenic Il17a⁺ and Ifng⁺ CD4⁺ T cells in the intestinal lamina propria but also by a marked elevation of Il10 in the Etv1‐deficient T cells, revealing a key immunoregulatory mechanism that contributes to the protective phenotype.

Another significant finding of our study is the context‐dependent role of Etv1 in immune regulation. Under steady‐state conditions, Etv1 deficiency did not disrupt immune homeostasis, and our pharmacological safety assessments further confirmed that inhibiting Etv1 with BRD32048 had no significant impact on liver function, kidney function, or baseline tissue integrity in non‐inflammatory settings. This strongly suggests a favorable therapeutic window for targeting Etv1. The key to this context‐specific function lies in its regulation. We found that T cell receptor (TCR) signaling acts as the primary switch for ETV1 expression, as evidenced by its time‐dependent upregulation upon anti‐CD3/CD28 stimulation. Intriguingly, this induction did not simply correlate with signal strength; higher levels of TCR stimulation did not lead to proportionally higher ETV1 expression. This nuanced pattern suggests that ETV1 may exert its primary function during phases of sustained, rather than maximally strong, T cell activation. This context‐dependent expression pattern has profound therapeutic implications. It indicates that an ETV1 inhibitor would preferentially target pathogenic T cells that are chronically activated within the inflammatory milieu, thereby effectively suppressing intestinal inflammation. Simultaneously, it would likely have minimal effect on naïve T cells or those involved in standard immune responses. Consequently, targeting ETV1 emerges as a promising strategy to achieve a favorable balance, suppressing pathological immune responses while preserving overall immune surveillance.

The amino acid metabolism program is crucial for CD4^+^ T cell activation, proliferation, and differentiation.^[^ ^33^, ^34^, ^35^, ^36^ ^]^ In these processes, multiple amino acid transporters are required to import basic building blocks to support the high levels of protein synthesis in T cells.^[^ ^37^, ^38^ ^]^ Solute carrier 7 family (Slc7) members are well studied amino acid transporters, which can be divided into the L‐type amino acid transporters (Slc7a5‐11, and Slc7a13), and the cationic amino acid transporters (Slc7a1‐Slc7a4, and Slc7a14). Among these members, Slc7a5 has been previously implicated in CD4^+^ T cell activation, proliferation, and Th17 cell differentiation.^[^ ^13^, ^14^ ^]^ Slc7a5 expression is greatly upregulated in CD4^+^ T cells after TCR stimulation,^[^ ^39^ ^]^ whereas the upstream transcriptional regulators of this process remained unknown. This study demonstrates a previously unrecognized function of Etv1 in regulating Slc7a5‐mediated amino acid uptake during CD4^+^ T cell responses. Etv1 deficient CD4^+^ T cells exhibit lower levels of Slc7a5, accompanied by defective amino acid uptake. Etv1 functions as a transcriptional factor that promotes Slc7a5 expression through binding to its gene promoter. Remarkably, forced expression of Slc7a5 reversed amino acid uptake, as well as CD4^+^ T cell proliferation and Th17 generation, in Etv1 deficient CD4^+^ T cells in vitro and restored the capability of the T cells to induce colitis in vivo. Thus, Slc7a5 is absolutely required for Etv1 in regulating CD4^+^ T cell proliferation, differentiation, and CD4^+^ T cell‐mediated intestinal inflammation.

Having established that Slc7a5 is required for Etv1 function, we evaluated its direct pharmacological inhibition using JPH203, a highly selective inhibitor of Slc7a5. JPH203 has previously demonstrated efficacy in preclinical models of autoimmunity, such as rheumatoid arthritis, psoriasis, and steroid‐resistant asthma, by specifically dampening pathogenic CD4⁺ T cell responses.^[^ ^40^, ^41^, ^42^ ^]^ Importantly, its favorable safety and tolerability profile has been established in early‐phase clinical trials for patients with advanced solid tumors.^[^ ^43^ ^]^ In our study, treatment with JPH203 significantly ameliorated CD4⁺ T cell‐mediated colitis in mice. This evidence confirms that therapeutic inhibition of Slc7a5 with JPH203 is effective and highlights the translational potential of repurposing Slc7a5 inhibition for IBD. Our work thus positions both ETV1 and Slc7a5 as nodes for intervention. Inhibiting the upstream transcription factor ETV1 may broadly suppress the pathogenic T cell program, whereas targeting Slc7a5 offers a more precise means to restrict the metabolic fuel for T cell expansion. The established clinical safety data for JPH203 provide a translational advantage. Therefore, determining whether upstream, downstream, or even combined targeting within this axis yields the optimal therapeutic profile represents a worthwhile direction for future research.

While our study establishes a crucial role for ETV1 in governing the pathogenicity of CD4⁺ T cells in IBD, several limitations should be considered. Our transcriptomic data indicate that Etv1 deficiency significantly alters the expression of a broad spectrum of genes, including those associated with B cell activation, mononuclear cell migration, and epithelial cell development (Figure 2B; Table S1, Supporting Information). This finding, coupled with our initial immunohistochemical observation of ETV1's broad cellular distribution in the inflamed colon, strongly implies that ETV1 likely exerts functions beyond CD4⁺ T cells. Although pharmacological inhibition of ETV1 was therapeutically effective in the T cell‐mediated colitis model, its potential impact on other cell types, such as intestinal epithelial cells, B cells, CD8⁺ T cells, and neutrophils, remains unknown. Therefore, future studies employing conditional knockout models are warranted to systematically delineate the cell‐intrinsic functions of ETV1 across these cellular compartments, which will be crucial for comprehensively evaluating its therapeutic potential and paving the way for targeted treatment strategies.

**Figure 9:**
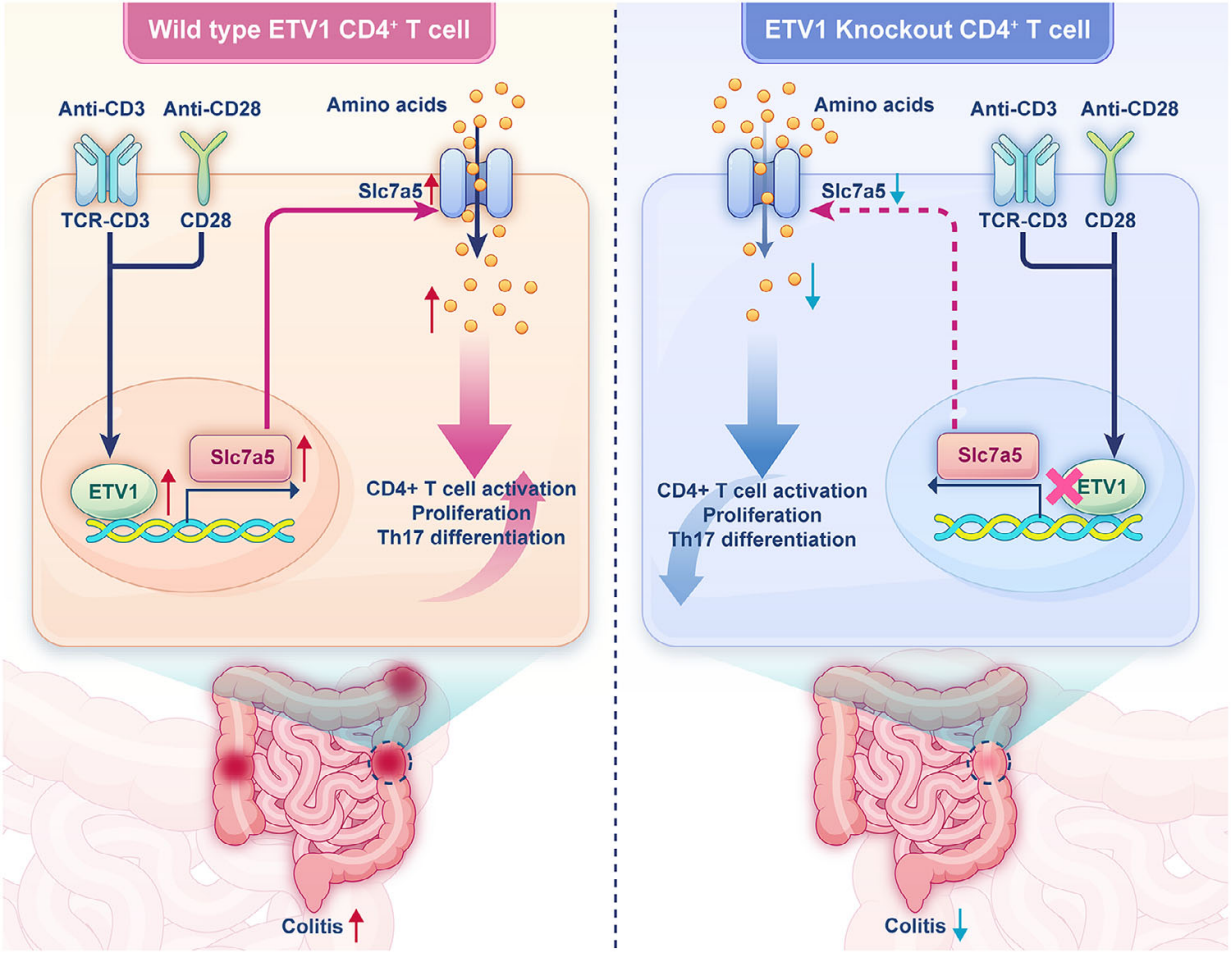
Mechanism of ETV1 in promoting T cell‐driven colitis. TCR stimulation induces ETV1, which drives Slc7a5‐dependent amino acid transport to metabolically potentiate CD4⁺ T cell activation, proliferation, and Th17 polarization. Targeting ETV1 effectively suppresses pathogenic T cell responses and intestinal inflammation.

In summary, our work provides the first evidence that ETV1 expression is significantly increased in active IBD patients and establishes the ETV1‐Slc7a5 axis as a critical driver of CD4⁺ T cell pathogenicity. We demonstrate that ETV1 controls CD4⁺ T cell‐mediated intestinal inflammation by regulating the amino acid transporter Slc7a5 (**Figure** **9**). Crucially, therapeutic targeting of this axis, either by inhibiting the upstream regulator ETV1 or the downstream metabolic effector Slc7a5, effectively ameliorates colitis in vivo. These findings establish the ETV1‐Slc7a5 axis as a highly promising target for the development of novel therapeutic strategies for IBD.

## 4 Experimental Section

### Subjects and Samples

All patients with IBD and healthy individuals enrolled in this research were recruited from the Digestive Endoscopic Center, Shanghai Sixth People's Hospital Affiliated to Shanghai Jiao Tong University School of Medicine (Shanghai, China) from March 2021 to August 2024. The diagnosis for patients with Crohn's disease (CD) or ulcerative colitis (UC) was confirmed by clinical signs and symptoms, endoscopic examination, and histological results after the exclusion of other diseases. Two colonoscopic biopsy tissues were collected from each patient with active UC (n = 17), patients with active CD (n = 13), and healthy controls (n = 17) and subjected to ETV1 and SLC7A5 qRT‐PCR, immunohistochemistry, and immunofluorescence analysis, respectively. Disease severity was assessed according to the Mayo index and CD activity index (CDAI) for patients with UC and CD, respectively. Endoscopic variables were scored by the Ulcerative Colitis Endoscopic Index of Severity (UCEIS) and the Simple Endoscopic Severity for CD (SES‐CD) as described previously.^[^ ^8^ ^]^ The clinical characteristics of patients with IBD are shown in Table S2, Supporting Information. This study was approved by the institutional review board of the Clinical Research of the Shanghai Sixth People's Hospital. Written informed consent was obtained from all participants before the study protocol.

### Mice

C57BL/6J background Etv1 knockout (Etv1 KO) mice, their wild type (WT) littermates, and Rag1^−/− ^mice were sourced from Gempharmatech Co., Ltd. (Nanjing, China). All mice were maintained in a specific pathogen‐free (SPF) facility at the animal center of Shanghai Chengxi Biotechnology Co., Ltd. [SCXK (Shanghai) 2023–0023]. The animal care and experiments were reviewed and approved by the Institutional Animal Care and Use Committee of Shanghai Chengxi Biotechnology Co., Ltd (Approval NO., CX052308077). All animals used were age‐ and sex‐matched in all experiments. All animal procedures were performed in accordance with the Regulations for the Administration of Laboratory Animals and the Guidelines for the Care and Use of Laboratory Animals. The 3R principles (replacement, reduction, and refinement) were strictly followed to minimize animal suffering and ensure animal welfare.

### Statistical Analysis

Statistical analyses were performed using GraphPad Prism 9.0 (GraphPad Software, San Diego, CA, USA). Data are presented as mean ± standard error of the mean (SEM). The sample size (n), representing the number of biologically independent replicates for each experiment, is provided in the figure legends. No specific data pre‐processing was applied unless otherwise stated, and all data points were included in the analysis without outlier removal. Comparisons between two groups were performed using an unpaired Student's t‐test. For comparisons among more than two groups, one‐way ANOVA was performed, followed by Dunnett's or Tukey's multiple comparison test as indicated in the figure legends. The Mann–Whitney U test was applied for the analysis of pathological scores. Correlations were assessed using Pearson's correlation analysis. Statistical significance was defined as p < 0.05, with the following annotations used in figures, ^✱^ *p* < 0.05, ^✱✱^ *p* < 0.01, ^✱✱✱^ *p* < 0.001, ^✱✱✱✱^ *p* < 0.0001; ns, not significant.

## Conflict of Interest

The authors declare no competing interests.

## Author Contributions

Y.S., S.W., and Y.Y. contributed equally to this work. Conceptualization, Y.S., X.W.; methodology, C.M., S.W., Y.Y.; investigation, Y.S., C.M., W.C., Y.C., D.C.; visualization, Y.S., Y.Y., W.C., Y.C.; funding acquisition, Y.S., C.M., Y.Y.; project administration, Y.S., C.M., X.W.; writing‐original draft, Y.S., Y.Y., C.M., S.W.; writing‐review & editing, Y.S., X.W.; Supervision, Y.S., C.M., X.W.

## Supporting information



Supporting Information

## Data Availability

The data that support the findings of this study are available from the corresponding author upon reasonable request.
