# Supplementary material for: ETV1 Drives CD4+ T Cell‐Mediated Intestinal Inflammation in Inflammatory Bowel Disease Through Amino Acid Transporter Slc7a5
Source: Adv Sci (Weinh). 2025 Dec 5;13(11):e11595. doi: 10.1002/advs.202511595 (PMC12931228; doi:10.1002/advs.202511595)
Supplement: Supplementary file 1 — Supporting Information [file ADVS-13-e11595-s001.docx]

**Supporting Information**

**Figure S1**


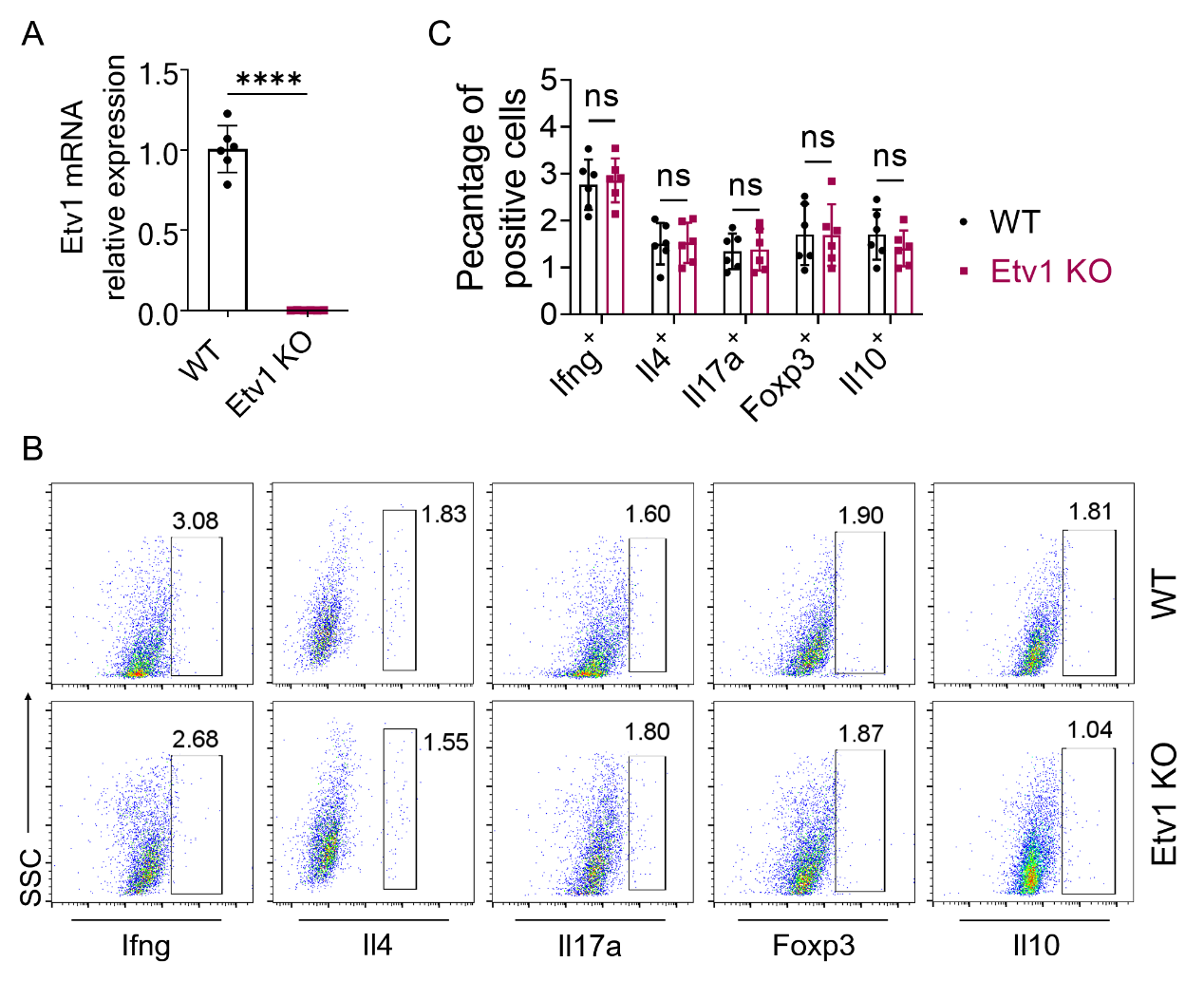


**Figure S1.** Cytokines expression in colonic CD4^+^ T cells from WT and Etv1 knockout (KO) mice (n = 6 per group). A) Validation of Etv1 knockout efficiency in colon tissues. ^✱✱✱✱^p < 0.0001. B, C) CD4^+^ T cells in intestinal lamina propria were isolated and purified using CD4 magnetic bead and the profile of Ifng^+^, Il4^+^, Il17a^+^, Foxp3^+^ and Il10^+^ cells were measured by flow cytometry. All data are pooled from three independent experiments and are expressed as mean ± SEM. Statistical analysis was evaluated using unpaired Student’s t-test. Il, interleukin; Ifn, interferon; Foxp3, forkhead box protein P3; ns, no significance.

**Figure S2**

**
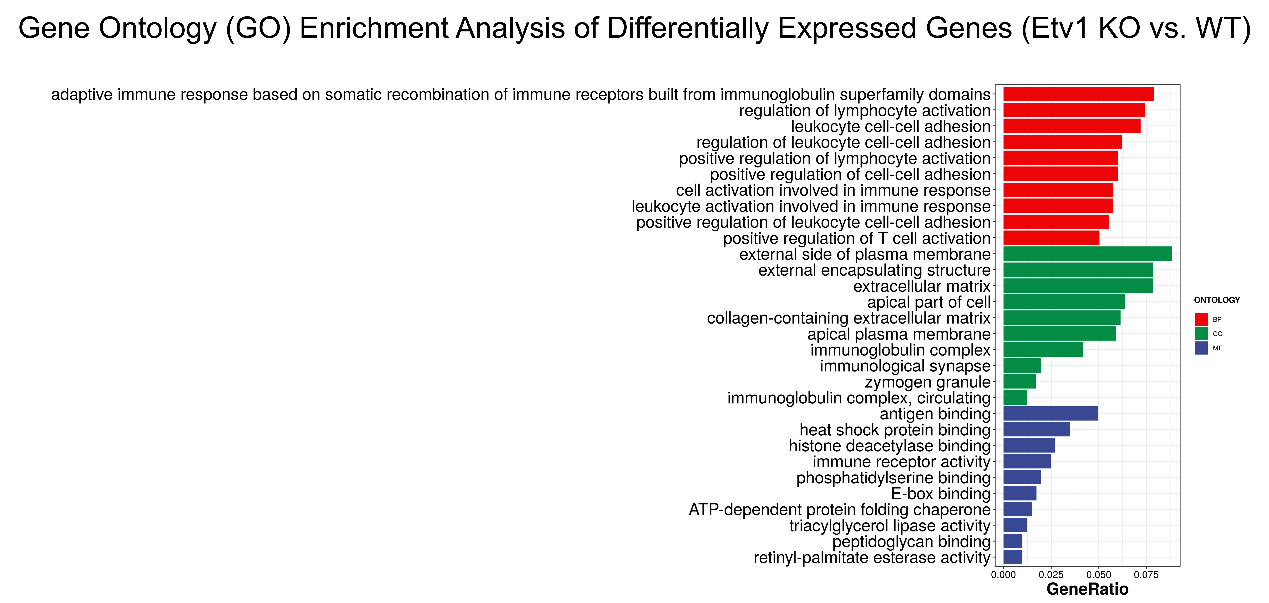
**

**Figure S2.** Gene Ontology (GO) enrichment analysis of differentially expressed genes from colon tissues of Etv1 KO and WT mice under steady-state conditions. The bar plot displays significantly enriched GO terms across biological process (BP, red), cellular component (CC, green), and molecular function (MF, blue) categories based on the differentially expressed genes identified in Figure 2A.

**Figure S3**


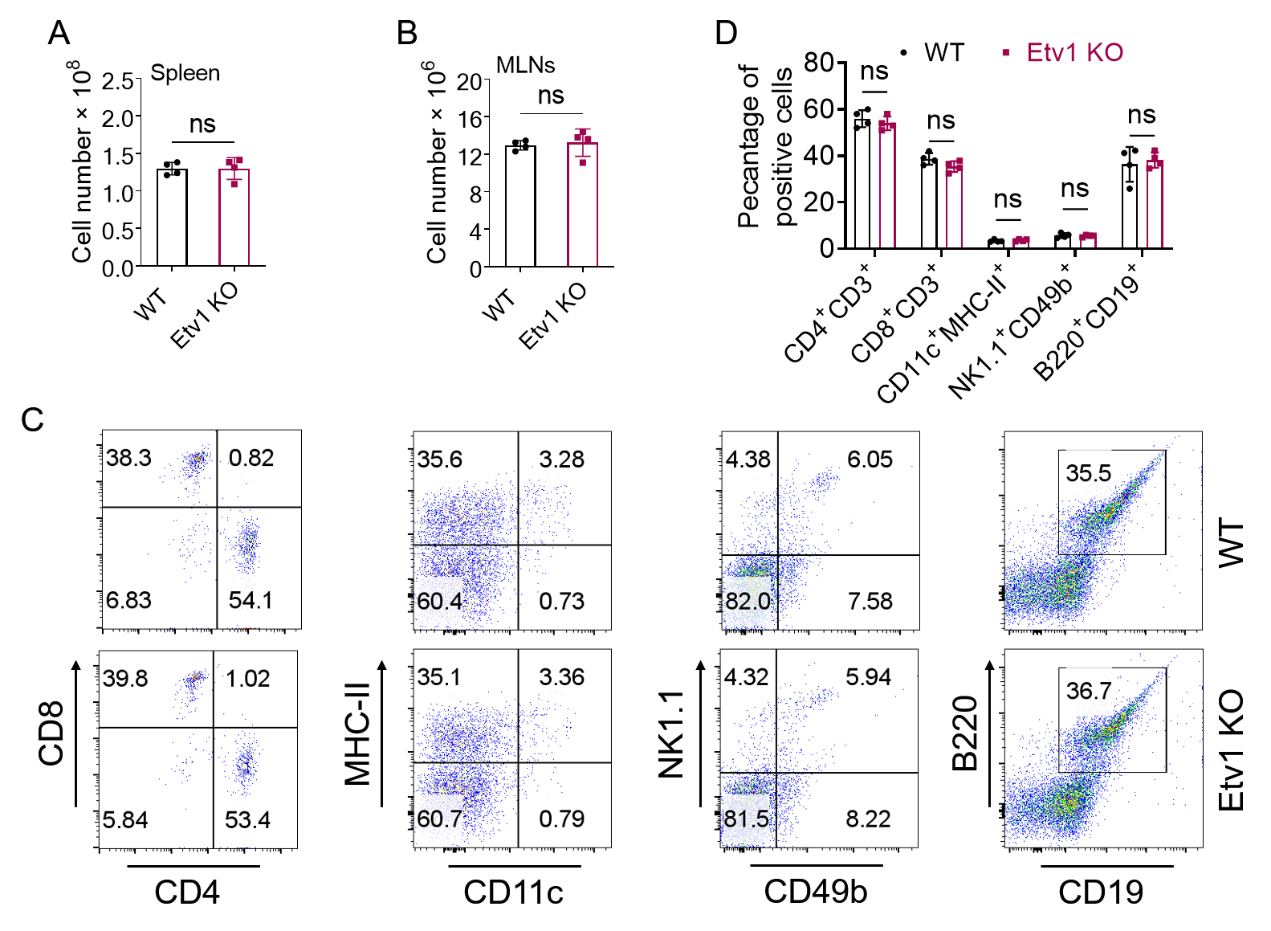


**Figure S3.** Immune homeostasis of Etv1 KO mice is normal. A, B) Total number of cells in spleens and mesenteric lymph nodes (MLNs) from 8-week-old WT and Etv1 KO mice (n = 4 per group). C) Representative flow cytometric images and D) frequencies of CD4^+^ and CD8^+^ T cells, CD11c^+^MHC-II^+^ DCs, NK1.1^+^CD49b^+^ NK cells and B220^+^CD19^+^ B cells in spleens from WT and Etv1 KO mice (n = 4 per group). ns, no significance. Data were expressed as mean ± SEM. Statistical analysis was evaluated using unpaired Student’s t-test.

**Figure S4**

**
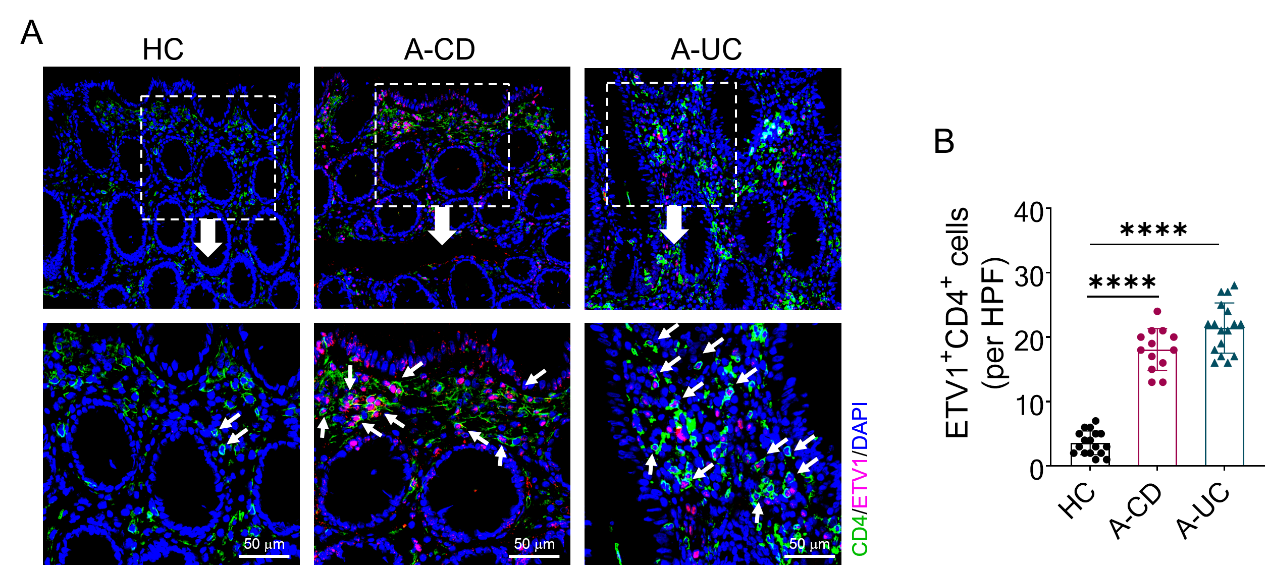
**

**Figure S4.** Infiltration of ETV1⁺CD4⁺ T cells in the inflamed intestinal mucosa. A) Immunofluorescence staining of ETV1 (red) and CD4 (green) on colonic sections from healthy controls (HC) and patients with ulcerative colitis (UC) or Crohn's disease (CD) (same cohort as in Figure 1A). Arrows indicate representative double-positive ETV1⁺CD4⁺ T cells. B) The number of ETV1⁺CD4⁺ T cells was quantified and is presented in the bar chart. ^✱✱✱✱^p < 0.0001. HC, healthy controls; A-CD, active Crohn’s disease; A-UC, active ulcerative colitis.

**Figure S5**

**
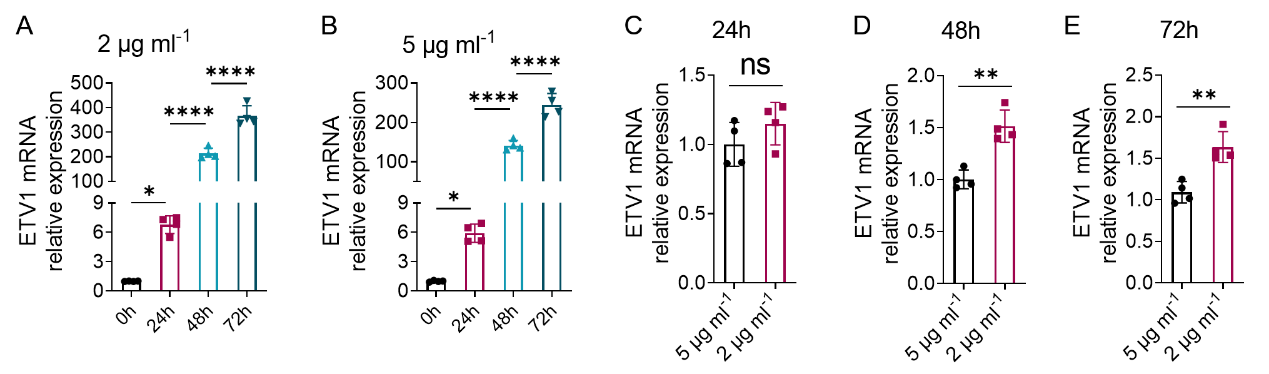
**

**Figure S5.** ETV1 expression is induced in human CD4⁺ T cells upon T cell receptor stimulation. Peripheral blood CD4⁺ T cells from healthy donors (n = 4) were activated with anti-CD3/CD28 antibodies for the indicated times or at the indicated concentrations. A) ETV1 mRNA expression in cells stimulated with anti-CD3 (2 µg mL^-1^) and anti-CD28 (2 µg mL^-1^) antibodies for the indicated times (0, 24, 48, 72 h). B) ETV1 mRNA expression in cells stimulated with anti-CD3 (5 µg mL^-1^) and anti-CD28 (5 µg mL^-1^) antibodies for the indicated times (0, 24, 48, 72 h). C-E) qRT-PCR analysis of ETV1 in cells stimulated with anti-CD3/CD28 antibodies at indicated doses for C) 24 h, D) 48h and E) 72h. ^✱^p < 0.05; ^✱✱^p < 0.01; ^✱✱✱✱^p < 0.0001.; ns, no significance. Statistical significance was determined by one-way ANOVA followed by Tukey's multiple comparisons test (A, B) or unpaired Student’s t-test (C-E).

**Figure S6**

**
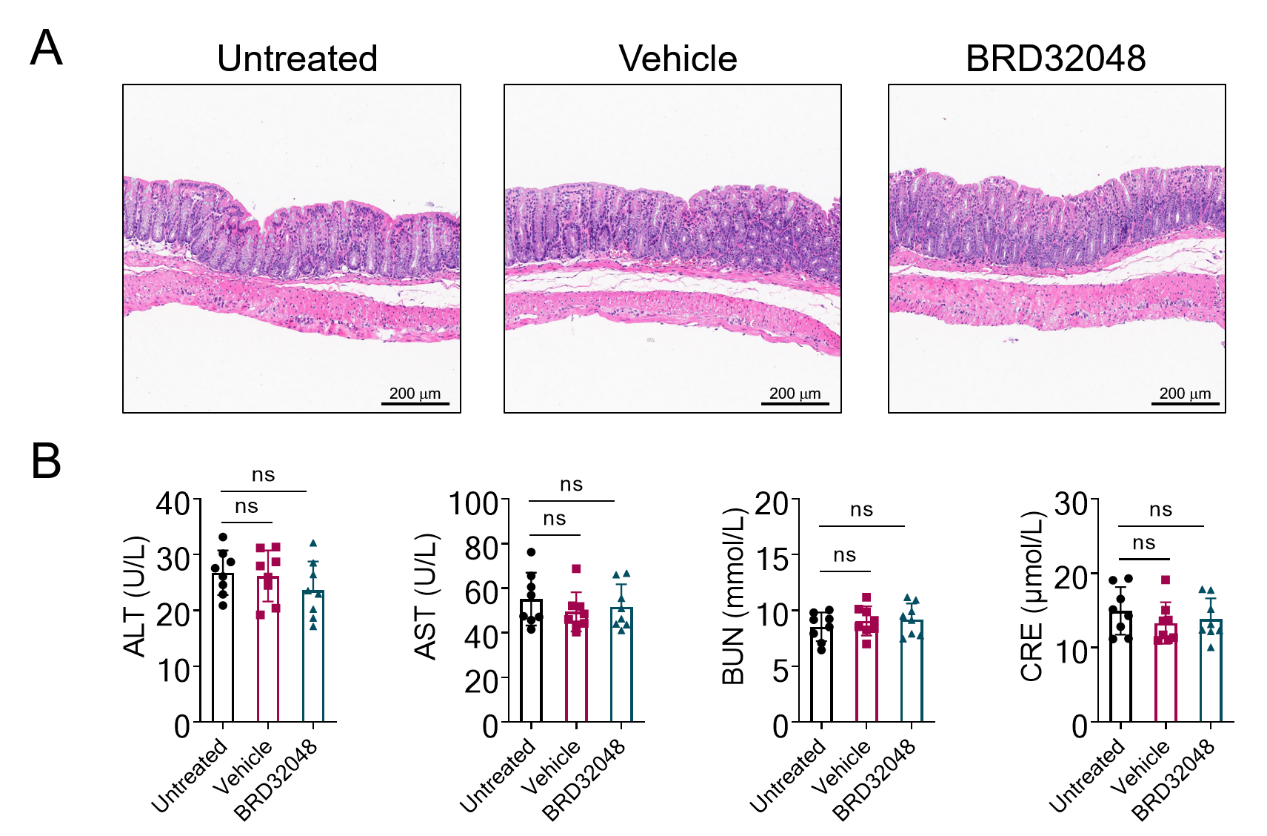
**

**Figure S6.** Evaluation of BRD32048 systemic toxicity in healthy WT mice. A) Representative H&E-stained images of colon sections from Untreated, Vehicle (DMSO), or BRD32048-treated mice. B) Serum biochemical analysis of liver and kidney function markers, including alanine aminotransferase (ALT), aspartate aminotransferase (AST), blood urea nitrogen (BUN), and creatinine (CRE), in mice from the indicated treatment groups. Data are presented as mean ± SEM (n = 8 mice per group). Statistical analysis was evaluated using one-way ANOVA followed by Dunnett’s multiple comparisons test. No significant differences were found between groups.

**Figure S7**

**
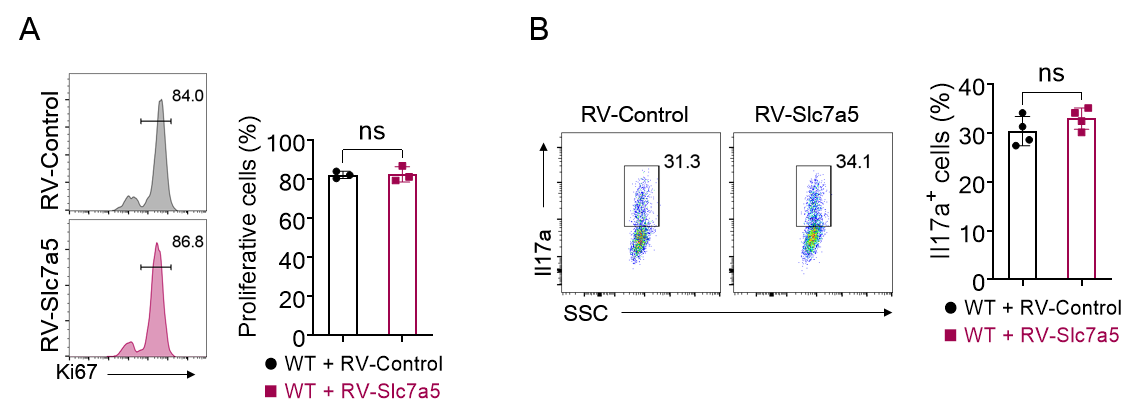
**

**Figure S7.** Forced expression of Slc7a5 has not obviously effect on WT CD4^+^ T cells proliferation and Th17 differentiation. A) WT CD4^+^ T cells transduced with control retrovirus (RV-Control) or Slc7a5-expressing retrovirus (RV-Slc7a5) were activated with anti-CD3/CD28 antibodies for 72h. Flow cytometric analysis of Ki67 staining. B) WT CD4⁺ T cells were transduced with RV-Control or RV-Slc7a5 and cultured under Th17-polarizing conditions (TGF-β + IL-6 + IL-23) for 4 days. Representative flow cytometric images (left) and the percentage (right) of Il17a^+^ cells. RV, retrovirus; ns, no significance.

**Experimental Section**

Antibodies and Cytokines: Monoclonal antibodies against mouse CD3/CD28 antibodies, human CD3/CD28 antibodies, anti-IL-4, anti-IFN-γ and LIVE/DEAD™ Fixable Near-IR Dead Cell Stain (L34976, dilution ratio 1:1000), ETV1 antibody, Fixation/Permeabilization solution and Permeabilization Buffer (00-5523-00) and TRIzol were purchased from Invitrogen. Brilliant Violet (BV) 510-conjugated anti-CD4, Phycoerythrin (PE)-conjugated anti-CD8, BV 421-conjugated anti-CD3, BV 650-conjugated IL-17A, BV 421-conjugated IL-10, FITC-conjugated IFN-γ, BV 605-conjugated IL-4, phycoerythrin (PE)-conjugated Foxp3 or PE-conjugated anti-mouse/human Ki-67, APC-conjugated anti-CD25, FITC-conjugated anti-CD69, PE-conjugated anti-CD98, LIVE/DEAD™ Fixable Near-IR Dead Cell Stain and cell staining buffer were from Biolegend. Recombinant mouse TGF-β, IL-23, IL-12 and IL-1β were from Novoprotein (Suzhou, China). Recombinant mouse IL-6 and IL-2 were from PeproTech (NJ, USA). Collagenase IV, Trinitrobenzene sulfonic acid (TNBS, Sigma, St. Louis, MO, USA), phorbol 12-myristate 13-acetate (PMA) and ionomycin were from Sigma-Aldrich. Slc7a5 (ab305251) were purchased from Abcam (Cambridge, UK).

Immunohistochemical Staining and Analysis of ETV1: Colonic biopsies from IBD patients and healthy controls were fixed in 10% formalin, paraffin-embedded, and sectioned at 5 μm. For ETV1 detection, sections were deparaffinized, rehydrated, and subjected to antigen retrieval using citrate buffer. After quenching endogenous peroxidase activity with 3% H₂O₂, sections were blocked with 10% normal goat serum. The sections were then incubated overnight at 4 °C with a mouse monoclonal anti-ETV1 primary antibody. Following PBS washes, bound antibody was detected using an HRP-conjugated goat anti-mouse secondary antibody and visualized with a DAB substrate kit, which produces a brown precipitate at the antigen site. Finally, sections were counterstained with hematoxylin, dehydrated, cleared, and mounted for examination under a bright-field microscope.

ETV1 immunoreactivity was semi-quantitatively assessed using a histochemistry scoring system, which integrates both the intensity of the staining and the percentage of positive cells. The staining intensity was graded as 0 (negative), 1 (weak, pale yellow), 2 (moderate, brownish-yellow), or 3 (strong, brown). The percentage of cells at each intensity level was estimated. The final histochemistry score (range 0-300) was calculated by multiplying the percentage of positive cells by their respective intensity grade, using the formula: histochemistry score = Σ (pi × i), where pi represents the percentage of cells with intensity i. A higher histochemistry score indicates a stronger combined positive signal, reflecting both the abundance and the intensity of ETV1 expression.

Immunofluorescence Staining: Immunofluorescence staining was conducted to concurrently localize ETV1/CD4 and SLC7A5/CD4 in human intestinal mucosal sections. Briefly, paraffin-embedded tissue sections from IBD patients and healthy controls were deparaffinized, rehydrated, and subjected to antigen retrieval. Sections were then permeabilized with 0.3% Triton X-100 in PBS for 10 minutes and blocked with 10% normal goat serum for 1 hour at room temperature. Subsequently, the sections were incubated overnight at 4 °C with specific primary antibody combinations. After washing, the sections were incubated with a mixture of fluorescent dye-conjugated secondary antibodies for 2 hours at room temperature in the dark. Cell nuclei were clearly identified by counterstaining with DAPI. Finally, images were under observed confocal microscope.

Isolation of Mouse CD4⁺ T Cells: CD4⁺ T cells were isolated from mouse mesenteric lymph nodes and spleens. The tissues were dissociated, and single-cell suspensions were prepared by filtration through a 100-μm strainer and erythrocyte lysis with Tris-NH₄Cl. Target cells were then positively selected by incubation with anti-mouse CD4 magnetic particles, followed by purification using a Cell Separation Magnet

In Vitro Activation, Proliferation and Differentiation of CD4^+^ T cells: For activation, splenic CD4^+^ T cells (5 × 10^5^/well) were stimulated with plate-coated anti-CD3 antibody (5 µg mL^-1^) and soluble anti-CD28 antibody (2 µg mL^-1^) in RPMI-1640 culture medium containing 10% fetal bovine serum (FBS), 1 mM sodium pyruvate, 50 μM 2-mercaptoethanol, 1% penicillin/streptomycin, 10 mM HEPES and 2mM glutamine. For proliferation assay, splenic CD4^+^ T cells (5 × 10^5^/well) were activated with anti-CD3/CD28 antibodies for 24, 48 or 72 h in vitro, and the number of Ki67^+^ CD4^+^ T cells was analyzed by flow cytometry. For T cell differentiation, splenic CD4^+^ T cells (5 × 10^5^/well) were stimulated with anti-CD3/CD28 antibodies and cultured simultaneously in the following conditions: Th1 (10 ng ml^-1^ IL-2, 10 ng ml^-1^ IL-12, 5 μg ml^-1^ anti-IL-4); Th2 (20 ng ml^-1^ IL-4, 5 μg ml^-1^ anti-IFN-γ); pathogenic Th17 (50 ng ml^-1^ IL-6, 10 ng ml^-1^ IL-23, 5 ng ml^-1^ TGF-β, 5 μg ml^-1^ anti-IL-4, 5 μg ml^-1^ anti-IFN-γ); non-pathogenic Th17 (50 ng ml^-1^ IL-6, 5 ng ml^-1^ TGF-β, 5 μg ml^-1^ anti-IL-4, 5 μg ml^-1^ anti-IFN-γ); Treg (5 ng ml^-1^ TGF-β, 10 ng ml^-1^ IL-2). After 5 days of cultivation, the differentiated T cells were re-stimulated for 4 h with PMA and ionomycin in the presence of Brefeldin A followed by intracellular staining of IFN-γ, IL-4, IL-17A, and Foxp3 to quantify the frequency of Th1, Th2, Th17, and Treg cells.

Retroviral Transduction: Retroviral transduction in CD4^+^ T cells was performed as previously described. Briefly, CD4^+^ T cells were separated from the spleens of 8-week-old male WT or ETV1 KO mice as described above and stimulated with anti-CD3/CD28 antibodies for 20 hours. These activated cells were cocultured with RV-Slc7a5 or RV-Control in the presence of polybrene (10 μg ml^-1^, Sigma-Aldrich), and were put in a flat angle centrifuge and centrifuged at 2500g for 2 hours at 30℃. Following incubation at 37°C for 3 hours, the viral supernatant was removed and replaced with fresh culture medium supplemented with polarizing cytokines and antibodies to facilitate CD4^+^ T cell proliferation and differentiation.

Flow Cytometry: For cell surface staining, cells from in vitro cultures or indicated tissues in vivo were directly incubated with antibodies and/or fixable live/dead near-IR dye in cell staining buffer at 4 ℃ for 30 min. For intracellular staining, cells were stimulated with PMA (50 ng ml^-1^), ionomycin (750 ng ml^-1^) and brefeldin A (1mg ml^-1^) for 5 hours in medium at 37 ℃. After stimulation, cells were stained with surface markers and then permeabilized and fixed with Fixation/Permeabilization solution for 30 min at 4 ℃, followed by intracellular staining for IL-17A, IFN-γ, IL-4, IL-10, Foxp3 or Ki67 in the permeabilization buffer after washing. Stained cells were detected by BD LSRFortessa flow cytometer and data was analyzed by FlowJo software (v10.8.1, Tree Star; Ashland, OR, USA). All samples and their comparisons in the same experiment were gated under the same parameters.

Induction of Trinitrobenzene Sulfonic Acid (TNBS)-Induced Colitis: As described previously, 8-10 weeks old WT and Etv1 KO mice were presensitized by 1% TNBS on day 1 and were injected intracolonically with 150 μL TNBS solution (2.5% TNBS in equal volume of ethanol) on day 8. Mice were weighed every day thereafter. On day 13, the spleens, LMNs and colon tissues were removed for further analysis.

Adoptive CD4^+^ T Cell Transfer Colitis: To induce adoptive transfer colitis, CD45RB^high^CD4^+^ T cells were separated from spleens of WT or Etv1 KO mice (8-10 weeks old) and intraperitoneally injected into Rag1^−/−^ recipient mice (5 × 10^5^/mice). Recipient mice were weighed weekly. Eight weeks after transfer, colon tissues were extracted for histological assessment, RNA extraction, immunofluorescence staining and intestinal mucosal lamina propria cells isolation.

Assessment of the Therapeutic Effect and In Vivo Safety of BRD32048: The therapeutic effect of BRD32048 was evaluated in the T cell transfer colitis model. Rag1^−/−^ recipient mice received WT CD45RBʰⁱᵍʰCD4⁺ T cells and were divided into two groups four weeks later. The treatment group was administered BRD32048 (20 mg kg^-1^, dissolved in DMSO) intraperitoneally every other day. The vehicle control group received an equivalent volume of DMSO alone. All mice were monitored for body weight changes weekly and were euthanized at week 8 for colon collection.

For safety assessment, wild-type mice were randomized into three cohorts: an untreated control, a vehicle control receiving DMSO, and a group treated with BRD32048 (20 mg kg^-1^) via intraperitoneal injection every other day for four weeks. At the endpoint, colon tissues were collected for histological analysis by H&E staining to assess tissue architecture. Simultaneously, serum was collected for biochemical analysis to evaluate liver and kidney function, measuring levels of alanine aminotransferase (ALT), aspartate aminotransferase (AST), blood urea nitrogen (BUN), and creatinine (CRE).

Preparation of Lamina Propria Cells: Intestinal lamina propria (LP) lymphocytes were separated as previously described. In brief, the colons were sliced into small pieces, washed with pre cooled PBS for 3 times, and incubated with 0.5 mM EDTA at 37˚C for 40 min. Then, stirred the tissues in 0.5 mg ml^-1^ collagenase IV at 37˚C for 40 min. The lamina propria cells were collected using a 100-μm cell filter (BD Falcon) and then purified on a 40/80% discontinuous Percoll gradient.

Hematoxylin/Eosin (H&E) staining and histological evaluation: Colonic tissue samples were removed and fixed in 4% paraformaldehyde at 4 ℃ for 24 h, embedded in paraffin, and then sliced and stained with H&E. The severity of tissue damage was blindly evaluated using a total histological score. This score was the sum of individual scores (ranging from 0 to 3) assigned for the following parameters: hyperplasia, goblet cell loss, crypt abscesses, ulceration, and inflammatory cell infiltration in the mucosa and submucosa.

RNA extraction and quantitative real-time PCR (qRT-PCR): Total RNA was extracted from cells and tissues using TRIzol reagent (Invitrogen) following the manufacturer’s protocol. RNA purity and concentration were determined spectrophotometrically. Subsequently, mRNA was reverse-transcribed into complementary DNA (cDNA) using the 5× PrimeScript RT Master Mix Kit (TaKaRa, Dalian, China) under standardized conditions. Gene expression analysis was performed using the TB Green Premix Ex Taq Kit (TaKaRa) on a QuantStudio 7 Flex Real-Time PCR System (Thermo Fisher Scientific). Amplification reactions were conducted in triplicate under the following thermal cycling conditions: 95°C for 30 sec, followed by 40 cycles of 95°C for 5 sec and 60°C for 30 sec. The relative mRNA expression levels of target genes were normalized to the endogenous reference gene GAPDH and quantified using the comparative 2^−ΔΔCT^ method. The primer sequences used in this study are listed in Supplementary Table 2**.**

RNA sequencing of steady-state colons: Colons from naïve Etv1 knockout (KO) and wild-type (WT) littermates were harvested under steady-state conditions (n = 3 biological replicates per genotype). Total RNA was extracted from the distal 1 cm of colon using TRIzol reagent (Invitrogen) according to the manufacturer's protocol. RNA purity and concentration were assessed using a NanoDrop™ One/OneC spectrophotometer, and RNA integrity was verified on an Agilent 4200 TapeStation system (RIN ≥ 7.0). Total RNA concentration was accurately quantified using Life Invitrogen Qubit® 3.0 fluorometer with the Qubit^TM^ RNA HS Assay Kit. Sequencing libraries were constructed as follows: Poly(A) + mRNA was enriched from total RNA using oligo(dT)-conjugated magnetic beads. The purified mRNA was fragmented, and first-strand cDNA was synthesized using random hexamer primers and M-MuLV reverse transcriptase. Second-strand cDNA was synthesized using DNA polymerase I and RNase H. The double-stranded cDNA was purified, end-repaired, A-tailed, and ligated with Illumina sequencing adapters. Fragments of approximately 200 bp were selected using AMPure XP beads, followed by PCR amplification and purification. Final library quality was assessed by quantifying concentration with Kapa qPCR and determining size distribution using an Agilent 4200 TapeStation system. Equimolar amounts of each library were pooled and sequenced on an Illumina NovaSeq 6000 platform with a PE150 strategy. Raw reads were processed with Fastp (v0.23.0) to remove adapter sequences and low-quality bases. Clean reads were aligned to the mouse reference genome (GRCm38) using HISAT2 (v2.1.0). Gene-level read counts were obtained with featureCounts (v2.0.3), and differential expression analysis was performed using DESeq2 (v1.18.1). Functional enrichment analysis of Gene Ontology (GO) pathways was conducted using clusterProfiler (v4.2.2).

Amino acid uptake assay: The amino acid uptake assay kit (#UP04-12, DOJINDO Laboratories, Kumamoto, Japan) was used to evaluate the amino acid uptake capacity of CD4^+^ T cells. In brief, CD4^+^ T cells from the spleens of Etv1 KO or WT mice (8 -12 weeks) were stimulated by anti-CD3/CD28 antibodies for 24 h. BPA was used as an amino acid analogue, and its uptake capacity was detected using a BPA-probe, which penetrates the cell membrane, binds to the BPA and emits strong fluorescence. The fluorescent intensity of the BPA with BPA-probe complex in the cells was determined.

| ONTOLOGY | ID | Description | GeneRatio | p.adjust | GeneID |
| --- | --- | --- | --- | --- | --- |
| BP | GO:0050863 | Regulation of T cell activation | 0.059952 | 0.000615 | Anxa1/Cd28/Cd4/Coro1a/Fcho1/Gimap3/H2-Ab1/H2-Eb1/Hsp90aa1/Hsph1/Il2rg/  Itgal/Lax1/Lgals9/Lrrc32/Nfkbiz/Ptpn22/Ptprc/Rac2/Rhoh/Shb/Spn/Spta1/Tnfrsf13c/  Zc3h12a |
| BP | GO:0030217 | T cell differentiation | 0.047962 | 0.00628 | Anxa1/Bcl11b/Cd28/Cd4/Cracr2a/Gimap3/Hsp90aa1/Ikzf1/Il18r1/Il2rg/Irf4/Lgals9/  Mr1/Nfkbiz/Ptpn22/Ptprc/Rhoh/Shb/Spn/Zc3h12a |
| BP | GO:0042129 | Regulation of T cell proliferation | 0.031175 | 0.02048 | Anxa1/Cd28/Cd4/Coro1a/Itgal/Lgals9/Lrrc32/Ptpn22/Ptprc/Rac2/Spn/Spta1/Tnfrsf13c/ |
| BP | GO:0072678 | T cell migration | 0.021583 | 0.003339 | Ccl20/Coro1a/Ecm1/Gpr15/Icam1/Itgal/Itgb7/Lgals9/Spn |
| BP | GO:0042113 | B cell activation | 0.043165 | 0.006501 | Cd180/Cd28/Cd79b/Dock10/Ighm/Igkj5/Ikzf1/Il2rg/Irs2/Lax1/Mef2c/Nfkbiz/Pik3cd/  Pou2af1/Ptprc/Shb/Tnfrsf13b/Tnfrsf13c |
| BP | GO:0042100 | B cell proliferation | 0.021583 | 0.013159 | Cd180/Ighm/Irs2/Mef2c/Nfkbiz/Ptprc/Shb/Tnfrsf13b/Tnfrsf13c |
| BP | GO:0045444 | Fat cell differentiation | 0.045564 | 0.001652 | Adgrf1/Arl4a/Creb1/Ffar4/Id4/Insig1/Klf5/Lpin1/Mmp11/Noct/Nr1d1/Pdgfra/Per2/  Pim1/Plac8/Srebf1/Steap4/Zbtb16/Zc3h12a |
| BP | GO:0071674 | Mononuclear cell migration | 0.033573 | 0.013229 | Anxa1/Ccl20/Coro1a/Cx3cr1/Cxcl14/Ecm1/Fut4/Gpr15/Icam1/Itgal/Itgb7/Lgals9/  S100a14/Spn |
| BP | GO:0002064 | Epithelial cell development | 0.033573 | 0.039984 | Adamtsl4/Arhgef26/Bcl11b/Bhlha15/Cldn1/Gsdmc2/Hif1a/Icam1/Id1/Klf5/Rilpl2/  Shroom3/Tfcp2l1/Vsig1 |

**Table S1. Gene lists for significantly enriched GO terms highlighting cellular programs presented in Figure 2B**

**Table S2. Clinical characteristics of subjects**

|  | **Biopsy samples** | | |  | **Blood samples** | | |
| --- | --- | --- | --- | --- | --- | --- | --- |
|  | **HC** | **CD** | **UC** |  | **HC** | **CD** | **UC** |
| **Number of patients** | 17 | 13 | 17 |  | 4 | 4 | 4 |
| **Age (years)** | 31.4±5.6 | 32.2±11.6 | 35.6±13.4 |  |  | 33.4±12.8 | 36.8±14.6 |
| **Gender** |  |  |  |  |  |  |  |
| Male | 8 | 5 | 7 |  | 2 | 2 | 2 |
| Female | 9 | 8 | 10 |  | 2 | 2 | 2 |
| **Disease duration (months)** |  | 40.4±11.6 | 34.8±12.4 |  |  | 42.4±15.8 | 38.2±16.8 |
| **Current therapy** |  |  |  |  |  |  |  |
| 5-aminosalicylates |  | 0 | 6 |  |  | 0 | 2 |
| Prednisolone acetate |  | 3 | 9 |  |  | 1 | 0 |
| Immunosuppressants |  | 0 | 0 |  |  | 0 | 0 |
| Biologics |  | 10 | 2 |  |  | 3 | 2 |
| **Disease extent (UC)*** |  |  |  |  |  |  |  |
| E1 |  |  | 4 |  |  |  | 1 |
| E2 |  |  | 8 |  |  |  | 2 |
| E3 |  |  | 5 |  |  |  | 1 |
| **Disease location (CD)*** |  |  |  |  |  |  |  |
| L1 |  | 0 |  |  |  | 0 |  |
| L2 |  | 6 |  |  |  | 2 |  |
| L3 |  | 7 |  |  |  | 2 |  |
| L4 |  | 0 |  |  |  | 0 |  |
| **CRP (mg/L)** |  | 36.4±12.4 | 34.2±13.6 |  |  | 38.8±15.4 | 36.7±14.8 |

*According to the Montreal classification system. HC, healthy controls; CD, Crohn’s disease; UC, ulcerative colitis

**Table S3. Primers for qRT-PCR used in this study**

| Mice gene | Forward | Reverse |
| --- | --- | --- |
| Il17a | TCAGCGTGTCCAAACACTGAG | CGCCAAGGGAGTTAAAGACTT |
| Ifng | GCCACGGCACAGTCATTGA | TGCTGATGGCCTGATTGTCTT |
| Il4 | GGTCTCAACCCCCAGCTAGT | GCCGATGATCTCTCTCAAGTGAT |
| Foxp3 | CACCTATGCCACCCTTATCCG | CATGCGAGTAAACCAATGGTAGA |
| Il10 | CTTACTGACTGGCATGAGGATCA | GCAGCTCTAGGAGCATGTGG |
| Etv1 | GTTTGTTCCAGACTATCAGGCTG | GGGCTGTGGGGTTCTTTCTT |
| Slc7a5 | ATATCACGCTGCTCAACGGTG | GCCGCCTGACTTGGAGATG |
| Gapdh | TGACCTCAACTACATGGTCTACA | CTTCCCATTCTCGGCCTTG |
| Human gene | Forward | Reverse |
| ETV1 | CTGAACCCTGTAACTCCTTTCC | AGACATCTGGCGTTGGTACATA |
| SLC7A5 | CCGTGAACTGCTACAGCGT | CTTCCCGATCTGGACGAAGC |
| GAPDH | GGAGCGAGATCCCTCCAAAAT | GGCTGTTGTCATACTTCTCATGG |
